# Novel stereoisomeric lignin-derived polycarbonates: towards the creation of bisphenol polycarbonate mimics†

**DOI:** 10.1039/d2py01523d

**Published:** 2023-01-17

**Authors:** Xianyuan Wu, Dan Xu, Mario De bruyn, Gregor Trimmel, Katalin Barta

**Affiliations:** a Stratingh Institute for Chemistry, University of Groningen Groningen The Netherlands katalin.barta@uni-graz.at; b Department of Chemistry, Organic and Bioorganic Chemistry, University of Graz Heinrichstrasse 28/II 8010 Graz Austria; c Institute for Chemistry and Technology of Materials (ICTM), NAWI Graz, Graz University of Technology Stremayrgasse 9 8010 Graz Austria

## Abstract

In this work, we have described a family of bio-based polycarbonates (PC-MBC) based on the unique lignin-derived aliphatic diol 4,4′-methylenebiscyclohexanol (MBC) that was sustainably sourced from lignin oxidation mixture. The detailed structure analysis of these polycarbonates has been confirmed by a series of 2D NMR (HSQC and COSY) characterizations. Depending on the stereoisomerism of MBC, the PC-MBC displayed a wide achievable *T*_g_ range of 117–174 °C and high *T*_d5%_ of >310 °C by variation of the ratio of the stereoisomers of MBC, offering great substitution perspectives towards a bisphenol-containing polycarbonates. Nonetheless, the most here presented PC-MBC polycarbonates were film-forming and transparent.

## Abstract 2

We have described a family of bio-based and property-tunable polycarbonates (PC-MBC) based on the novel stereoisomeric lignin-derived aliphatic diol 4,4′-methylenebiscyclohexanol (MBC) that was sustainably sourced from lignin oxidation mixture.

## Graphical abstract

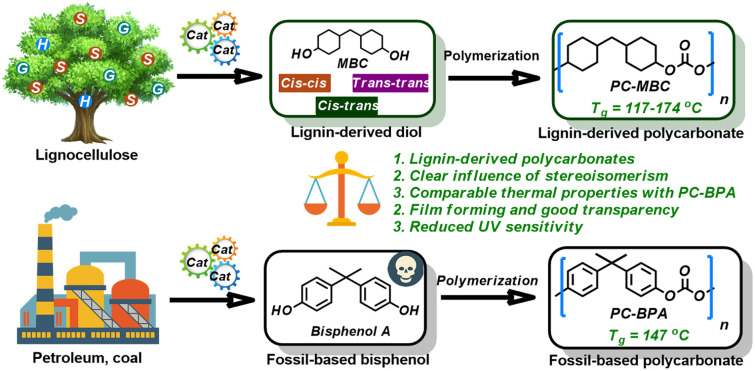

Polycarbonates (PC) are an important class of polymers, which over recent years have found broad applications towards a wide range of topical fields such as electronics, construction materials, 3D printing, automotive, aircraft, and (bio)medical applications.^1–4^ Central to this are their excellent thermal and mechanical properties, impact resistance, and optical features.^4^ Originally, polycarbonates were obtained by reacting diols with phosgene, a notoriously toxic gas. Also, this process uses vast amounts of methylene chloride solvent and tends to lead to the formation of stoichiometric amounts of chlorine salts. Aside this technology, a range of alternatives were developed notably (a) melt transesterification and polycondensation of a diol with a suitable carbonate;^5,6^ (b) the copolymerization of carbon dioxide (CO_2_) with an epoxide;^7,8^ (c) polymerization of suitable polyol diallyl carbonates^9^ and (d) ring-opening polymerization (ROP) of cyclic carbonate monomers.^10–12^

Bisphenol A (BPA) polycarbonate (PC) is a very important polymer material as it displays excellent thermal properties characterized by a *T*_g_ of 147 °C.^13,14^ Also, it is well known for its excellent miscibility, transparency, processability, and durability – all characteristics which largely relate to its stable amorphous structure.^15^ However, it has also been revealed that BPA-PC is sensitive to (UV-)light and hydrothermal aging, which makes for a small yet steady release of BPA monomer in the environment.^16^ This has been a concern as BPA has been identified as an endocrine disrupting chemical.^17^ Additionally, it has been shown that the presence of higher BPA levels carries, among others, a higher risk of heart disease, diabetes, and elevated liver enzymes.^18,19^ Recent years has seen the development of a range of BPA alternatives such as BPF [formaldehyde (F)-based], BPAF (hexafluoroacetone-based) (BPAF) and BPS (sulfur trioxide – based).^20,21^ To date the latter compound, commonly known as bisphenol S, is the most applied bisphenol A analogue in so called BPA-free products, while it displays a higher heat and photo resistance.^20^ However, scientific studies are increasingly showing that all these bisphenol analogues are little benign themselves, and sometimes even equally harmful than the original BPA.^20,22^ This all makes that a high demand exists for the development of truly benign bisphenol A alternatives and this preferentially from renewable resources such as lignocellulosic biomass.^23,24^ To date the most common bio-based BPA-alternative is based on the use of bisguaiacol (BG) instead of bisphenol (BP).^13,25–27^ Most revealing, depending on the exact BG regioisomer, a lower to non-existent estrogen activity is being observed. Additionally, BG-based polycarbonates display similar thermal and mechanical properties than BP-based ones.^28^ However, alternatives for BP-based polycarbonates with high *T*_g_ such as encountered with PC-BPA (*T*_g_,147 °C) remain to date somewhat elusive.^13^ Other bio-based polycarbonates with a wide range of thermal and mechanical properties have also been developed, the central molecular unit typically being epoxides, bisphenols or diols.^21^ Some illustrative examples are poly(1,4-cyclohexadiene oxide carbonate) (*T*_g_ ∼ 115 °C),^29^ poly(limonene-8,9-oxide carbonate) (*T*_g_: ∼140 °C),^30^ poly(limonene carbonate) (*T*_g_: ∼130 °C),^31^ and poly(isosorbide carbonate) (*T*_g_ ∼ 170 °C).^32^

The effect of stereoisomerism on the properties of polymers is presently still a lesser investigated topic.^33^ To date the main effect of changing the *cis*–*trans* isomerism of cyclohexane rings in polymer backbones regards changes in crystallinity.^34^ This is though a most important feature, as differences in the degree of crystallinity strongly influence the thermal and mechanical properties.^34^ Exemplary is the case of 1,4-cyclohexane-dimethanol (14CHDM) enriched PET, where addition of *trans* or *cis* 14CHDM is shown to markedly enhance the thermal properties and even induce crystallinity or amorphism.^35^

In this work, we report a range of novel bio-based, polycyclic, non-aromatic polycarbonates to which the *T*_g_ varies between 117 and 174 °C by variation of the ratio of the stereoisomers of the central structural monomer: lignin-derived 4,4′-methylenebiscyclohexanol (MBC). Importantly, this range covers the *T*_g_ values of a wide range of bisphenol-based polycarbonates. Most of the MBC-based polycarbonates are film-forming and transparent. To the MBC monomer, our group previously reported an elegant synthesis starting from lignin-derived compounds.^36,37^ (see also Fig. 1A).

**Fig. 1:**
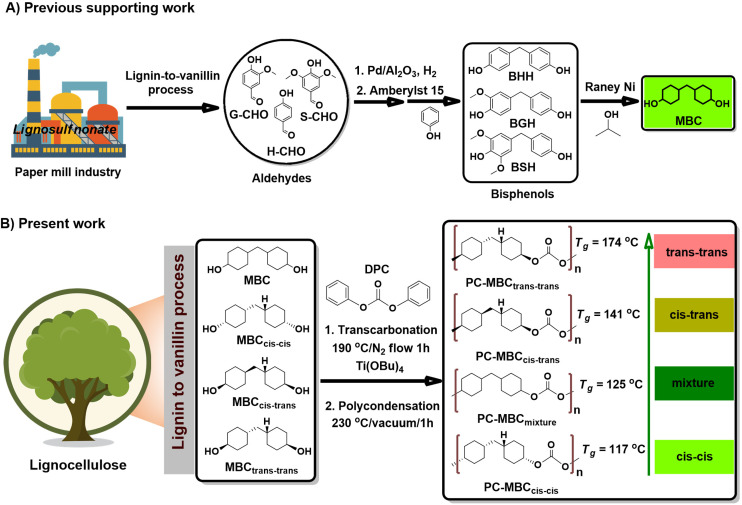
(A) Previous work by the group on the synthesis of MBC diol from product mixtures derivable from the lignosulfonate-to-vanillin process; (B) Influence of the MBC stereoisomerism on specifically the glass transition temperature of PC-MBC polycarbonates prepared by copolymerization of MBC, or any of its pure isomers, with diphenyl carbonate (DPC).

All here synthesized polycarbonates (PC-MBC) were prepared in accordance with a literature reported one-pot, two-step melt polymerization process (Fig. 1B).^6^ Their structural analysis was performed by FT-IR (Fig. 3A) and NMR spectroscopy (^1^H NMR, ^13^C NMR, 2D HSQC and 2D COSY) (see ESI Note 3†). More specifically, FT-IR characterization revealed the clear absence of the MBC-OH stretching vibration at 3250 cm^−1^ as well as the presence of a carbonate carbonyl stretching vibration band at 1730 cm^−1^, thus confirming the successful copolymerization of MBC with diphenyl carbonate (DPC) to yield PC-MBC. Additionally, ^1^H NMR spectroscopy showed the downfield shift of the ^1^H C**H̲**–OH signals of MBC at 3.54 ppm (*cis*–*cis* & *cis*–*trans*) and 3.94 ppm (*cis*–*trans* & *trans*–*trans*) to respectively 4.5 and 4.8 ppm (Fig. 2A), which is evidence of the formation of a C**H̲**–C=O group.^37^ Furthermore, with the numerical integration of the ^1^H C**H̲**–C=O peaks of PC-MBC being no different from the one of the MBC ^1^H C**H̲**–OH peaks (ratio: 1 : 2.4), non-preferential insertion of the MBC stereoisomers into the polymer chain can be inferred (Fig. S3†). Convincingly, the integration value of MBC's bridging methylene protons in PC-MBC shows nearly the same ratio of (11 : 42 : 47) (Fig. 3D) as with the original MBC stereoisomer mixture (Fig. 3E).

**Fig. 2:**
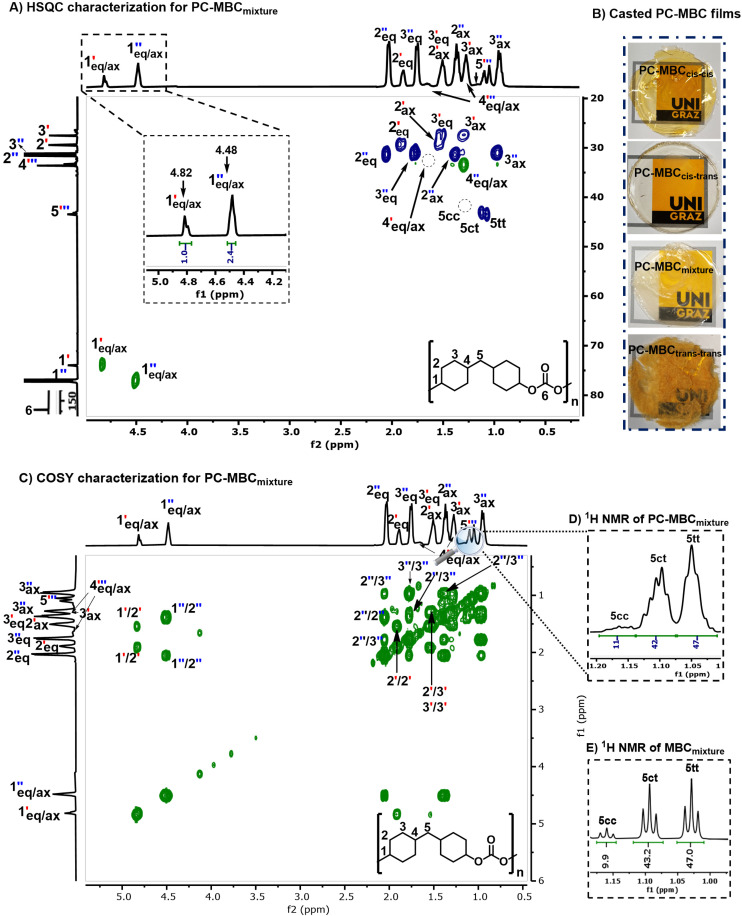
Structural eludication of PC-MBC_mixture_. (A) 2D-HSQC characterization of PC-MBC_mixture_. (B) Casted PC-MBC films. (C) 2D-COSY characterization of PC-MBC_mixture_. (D) Determination of the MBC isomer ratio in regular ^1^H NMR of PC-MBC_mixture_ by means of MBC's bridging methylene protons. (E) Determination of the MBC isomer ratio in regular ^1^H NMR by means of MBC's bridging methylene protons.

**Fig. 3:**
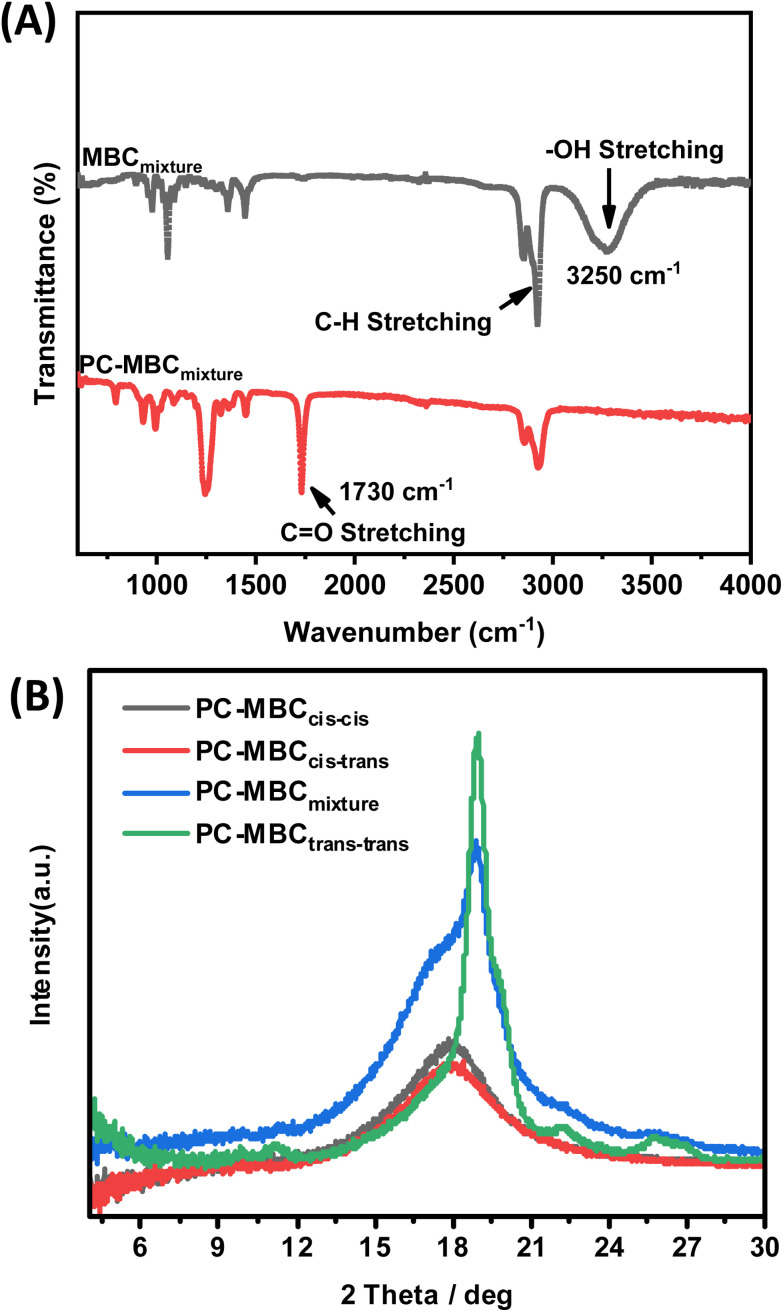
Structural eludication of PC-MBC. (A) FTIR spectroscopy of MBC and PC-MBC_mixture_. (B) XRD patterns of PC-MBC_*cis*–*cis*_, PC-MBC_*cis*–*trans*_, PC-MBC_*_mixture_*_ and PC-MBC_*trans*–*trans*_.

The polymeric properties of the PC-MBC polycarbonates were gauged by GPC analysis and DSC/TGA. GPC revealed *M*_w_ values between ∼23 000 g mol^−1^ for PC-MBC_mixture_ (stereoisomer ratio 11 : 42 : 47) and ∼28 000 g mol^−1^ for PC-MBC_*cis*–*trans*_, and PC-MBC_*cis*–*cis*_. PC-MBC_*trans*–*trans*_ exhibits a somewhat lower value of 19 200 g mol^−1^ due to the presence of oligomers (Fig. S44†). DSC analysis revealed a profound effect of the MBC stereoisomerism on the thermal properties. And indeed, per Table 1 the *T*_g_ of PC-MBC_*cis*–*cis*_ is with 117 °C significantly lower than the ones obtained for PC-MBC_*cis*–*trans*_ (141 °C) and PC-MBC_*trans*–*trans*_ (174 °C) and this despite PC-MBC_*trans*–*trans*_ displaying the lowest molecular weight. The higher *T*_g_ value for PC-MBC_*trans*–*trans*_ is in line with the existing literature which tends to link higher *T*_g_ values to higher *trans* contents.^38–43^ The *T*_g_ of PC-MBC_mixture_ (stereoisomer ratio 11 : 42 : 47) takes an intermediate value of 125 °C, which upon variation of the MBC stereoisomer ratios can be likely broadly varied in the 117–174 °C window. It is noteworthy that the *T*_g_ value of PC-MBC_*cis*–*trans*_ (141 °C) is very close to the one of PC-BPA (147 °C for a *M*_w_ of 126 kDa and 134 °C for a *M*_w_ of 16 kDa).^13^ Additionally, the *T*_g_ of PC-MBC_*cis*–*cis*_ (117 °C) is near equal to the one of PC-BPF (114 °C) and the *T*_g_ of PC-MBC_mixture_ (125 °C) mimics the one of PC-BGA (126 °C).^13^ Very importantly, tailored targeting of the *T*_g_ value of PC-MBC to the ones of other bisphenol/bisguaiacol polycarbonates, by variation of the MBC stereoisomer ratios, is a realistic option.

**Table 1:** Compilation of the molecular-weight distributions and thermal properties for the here presented PC-MBC polycarbonates^a^

Entry	Products	Yield^b^ [%]	*M*_w_ c [g mol^−1^]	*M*_n_ c [g mol^−1^]	*Đ*	*T*_m_ e [°C]	*T*_d_ d [°C]	*T*_g_ e [°C]
1	PC-MBC_*cis*–*cis*_	80.5	28 900	16 000	1.81	N/A	319	117
2	PC-MBC_mixture_ stereoisomer ratio (11 : 42 : 47)	87.4	22 700	12 600	1.8	N/A	326	125
3	PC-MBC_*cis*–*trans*_	84.0	28 300	18 300	1.54	N/A	322	141
4	PC-MBC_*trans*–*trans*_	79.0	19 200	7900	2.44	212	331	174

a Reaction conditions: 2.5 mmol MBC, 2.5 mmol DPC, 1 mol% titanium(iv) butoxide (TBT) catalyst, 190 °C N_2_/1 h, 230 °C/1 h under vacuum 1 mba.

b Yield (%) = weight of collect product/weight of theoretical product×100%.

c Molecular weight distribution was determined by GPC.

d *T*_d_ = temperature of decomposition – as determined by TGA characterization.

e *T*_g_ = glass transition temperature and T_m_ = melting temperature were determined by DSC characterization.

It is noteworthy that XRD analysis revealed that the main feature of all PC-MBC polymers is a halo at a 2*θ*-value of 18°, a characteristic feature for amorphous materials (Fig. 3). However, PC-MBC_*trans*–*trans*_ has a distinct crystalline fraction represented by a series of reflections making it thus semicrystalline. This is also confirmed by DSC as it is the sole polymer with a small melting point at 212 °C (Table 1). The PC-MBC_mixture_ [(stereoisomer ratio 11 : 42 : 47)] also shows a small reflection indicating the presence of a very small crystalline fraction. Given though the low degree of crystallinity of PC-MBC_*trans*–*trans*_, it is realistic to assume that fast cooling from the melt could yields a predominantly amorphous material. All this is in accordance with other types of polycarbonates being amorphous, the exception being some stereospecific polycarbonates.^44^ Explanatory to our observations could be the rigidity of the MBC molecules and the tendency of especially MBC_*trans*–*trans*_ to engage in intermolecular interactions. For all here evaluated PC-MBC polymers, the decomposition temperature (*T*_d_) is well above 300 °C, the highest recorded value being 331 °C for PC-MBC_*trans*–*trans*_ (Table 1).

In conclusion, we have demonstrated strong influences of stereoisomerism on the crystallinity and thermal properties of a range of MBC-based polycarbonates. More specifically, depending on the MBC stereoisomerism (ratio), the *T*_g_ value of the polymer can be tuned within a remarkably wide temperature range [117 to 174 °C] and high *T*_d5%_ of >310 °C. Importantly, most here presented MBC-based polycarbonates are film-forming and transparent, this holding real potential to the possible substitution of a wide range of polycarbonates such as PC-BPA, PC-BGA and PC-BPF. Also, due to the presence of only aliphatic cyclic structures in the polymeric backbone, the here presented PC-MBC polycarbonates are expected to be less susceptible to degradation by UV-light, which would constitutes an important advantage over PC-BPA and other bisphenol/bisguaiacol-containing polycarbonates. Future work should focus on scaling of the MBC synthesis strategies as well as optimizing of the purification protocols to allow easy access to all MBC isomers in pure form, and the corresponding polycarbonates. With these materials in hand, the polycarbonates should be subjected to further testing of UV resistance, as well as in-depth evaluation toward industrially relevant applications.

## Conflicts of interest

There are no conflicts of interest to declare.

## Supplementary Material

PY-014-D2PY01523D-s001

## References

[cit1] LeGrand D. G. and Bendler J. T., Handbook of Polycarbonate Science and Technology, Marcel Dekker, Inc., 2000

[cit2] Feng J., Zhuo R. X., Zhang X. Z. (2012). Prog. Polym. Sci..

[cit3] Cantrell J. T., Rohde S., Damiani D., Gurnani R., DiSandro L., Anton J., Young A., Jerez A., Steinbach D., Kroese C., Ifju P. G. (2017). Rapid Prototyp. J..

[cit4] Brunelle D. J. (2005). ACS Symp. Ser..

[cit5] Vanderhenst R., Miller S. A. (2013). Green Mater..

[cit6] Wu Y. H., Wang C. C., Chen C. Y. (2020). J. Polym. Res..

[cit7] Zhu Y. Q., Romain C., Williams C. K. (2016). Nature.

[cit8] Hauenstein O., Agarwal S., Greiner A. (2016). Nat. Commun..

[cit9] Carl J. C. and Haynes R. L., EP0144782A2, 1985

[cit10] Yu W., Maynard E., Chiaradia V., Arno M. C., Dove A. P. (2021). Chem. Rev..

[cit11] Suriano F., Coulembier O., Hedrick J. L., Dubois P. (2011). Polym. Chem..

[cit12] Tezuka K., Komatsu K., Haba O. (2013). Polym. J..

[cit13] Koelewijn S. F., Ruijten D., Trullemans L., Renders T., Van Puyvelde P., Witters H., Sels B. F. (2019). Green Chem..

[cit14] Hoeks T., Goossens J., Vermeulen H., Shaikh A. A. G. (2022). Polym. Eng. Sci..

[cit15] Hoekstra E. J., Simoneau C. (2013). Crit. Rev. Food Sci. Nutr..

[cit16] https://backend.orbit.dtu.dk/ws/portalfiles/portal/110762088/BPA_MST_project_No_1710_2015.pdf

[cit17] Rubin B. S. (2011). J. Steroid Biochem..

[cit18] Ma Y., Liu H. H., Wu J. X., Yuan L., Wang Y. Q., Du X. D., Wang R., Marwa P. W., Petlulu P., Chen X. H., Zhang H. Z. (2019). Environ. Res..

[cit19] Tarafdar A., Sirohi R., Balakumaran P. A., Reshmy R., Madhavan A., Sindhu R., Binod P., Kumar Y., Kumar D., Sim S. J. (2022). J. Hazard. Mater..

[cit20] Thoene M., Dzika E., Gonkowski S., Wojtkiewicz J. (2020). Nutrients.

[cit21] Liguori F., Moreno-Marrodan C., Barbaro P. (2020). Chem. Soc. Rev..

[cit22] Rochester J. R., Bolden A. L. (2015). Environ. Health Perspect..

[cit23] Cui S., Borgemenke J., Qin Y., Liu Z., Li Y. (2019). Adv. Bioenergy.

[cit24] Nguyen H. T. H., Qi P. X., Rostagno M., Feteha A., Miller S. A. (2018). J. Mater. Chem. A.

[cit25] Koelewijn F., Van den Bosch S., Renders T., Schutyser W., Lagrain B., Smet M., Thomas J., Dehaen W., Van Puyvelde P., Witters H., Sels B. F. (2017). Green Chem..

[cit26] Reno K. H. , Joseph Francis Stanzione I., Wool R. P., Sadler J. M., LaScala J. J. and Hernandez E. D., US10723684B2, 2015

[cit27] Harvey B. G., Guenthner A. J., Meylemans H. A., Haines S. R. L., Lamison K. R., Groshens T. J., Cambrea L. R., Davis M. C., Lai W. W. (2015). Green Chem..

[cit28] Peng Y., Nicastro K. H., Epps T. H., Wu C. Q. (2018). J. Agric. Food Chem..

[cit29] Winkler M., Romain C., Meier M. A. R., Williams C. K. (2015). Green Chem..

[cit30] Li C. L., Veldhuis T., Reuvers B., Sablong R. J., Koning C. E. (2020). Polym. Int..

[cit31] Hauenstein O., Reiter M., Agarwal S., Rieger B., Greiner A. (2016). Green Chem..

[cit32] Gomez Miranda-Jimenez Aberasturi O., Centeno-Pedrazo A., Fernandez S. P., Alonso R. R., Medel S., Cuevas J. M., Monsegue L. G., De Wildeman S., Benedetti E., Klein D., Henneken H., Ochoa-Gomez J. R. (2021). Green Chem. Lett. Rev..

[cit33] Liu J. H., Zhang Y., Phan H., Sharenko A., Moonsin P., Walker B., Promarak V., Nguyen T. Q. (2013). Adv. Mater..

[cit34] Worch J. C., Prydderch H., Jimaja S., Bexis P., Becker M. L., Dove A. P. (2019). Nat. Rev. Chem..

[cit35] Wang J. G., Liu X. Q., Jia Z., Sun L. Y., Zhang Y. J., Zhu J. (2018). Polymer.

[cit36] Wu X. Y., Galkin M. V., Barta K. (2021). Chem. Catal..

[cit37] Wu X. Y., De bruyn M., Trimmel G., Zangger K., Barta K. (2022). ACS Sustainable Chem. Eng..

[cit38] Vanhaecht B., Rimez B., Willem R., Biesemans M., Koning C. E. (2002). J. Polym. Sci., Part A: Polym. Chem..

[cit39] Celli A., Marchese P., Sisti L., Dumand D., Sullalti S., Totaro G. (2013). Polym. Int..

[cit40] Celli A., Marchese P., Sullalti S., Berti C., Barbiroli G. (2011). Macromol. Chem. Phys..

[cit41] Berti C., Celli A., Marchese P., Marianucci E., Barbiroli G., Di Credico F. (2008). Macromol. Chem. Phys..

[cit42] Berti C., Celli A., Marchese P., Marianucci E., Sullalti S., Barbiroli G. (2010). Macromol. Chem. Phys..

[cit43] Vanhaecht B., Teerenstra M. N., Suwier D. R., Willem R., Biesemans M., Koning C. E. (2001). J. Polym. Sci., Part A: Polym. Chem..

[cit44] Liu S. J., Wang X. H. (2017). Curr. Opin. Green Sustainable Chem..

