# Supplementary material for: Novel stereoisomeric lignin-derived polycarbonates: towards the creation of bisphenol polycarbonate mimics
Source: Polym Chem. 2023 Jan 17;14(8):907–12. doi: 10.1039/d2py01523d (PMC9942097; doi:10.1039/d2py01523d)
Supplement: PY-014-D2PY01523D-s001 [file PY-014-D2PY01523D-s001.pdf]

Electronic Supplementary Material (ESI) for Polymer Chemistry.
This journal is © The Royal Society of Chemistry 2023

Supplementary information

Novel stereoisomeric lignin-derived polycarbonates: towards the creation of bisphenol polycarbonate mimics

Xianyuan Wu[a], Dan Xu[b], Mario De bruyn[b], Gregor Trimmel[c], and Katalin Barta[a,b] *

[a]Stratingh Institute for Chemistry, University of Groningen, Groningen, The Netherlands.

[b]Department of Chemistry, Organic and Bioorganic Chemistry, University of Graz, Heinrichstrasse 28/II, 8010 Graz, Austria

[c]Institute for Chemistry and Technology of Materials (ICTM), NAWI Graz, Graz University of Technology, Stremayrgasse 9, 8010 Graz, Austria

* E-mail: katalin.barta@uni-graz.at

Table of Contents

Supplementary Note 1. General information 1

1.1 Materials and reagents 2

1.2 Preparation of the model compounds 2

1.3 Conversion, selectivity and yield calculation 2

1.4 General experimental procedures 3

Supplementary Note 2. Analysis and characterizations of MBC and its isomers 4

2.1 NMR spectra of MBC and its isomers 4

2.2 NMR-based structural determination of the cis/trans configurations of the pure MBC isomers 10

Supplementary Note 3. Analysis and characterizations of PC-MBC$_{cis\text{-}cis}$, PC-MBC$_{cis\text{-}trans}$, PC-MBC$_{trans\text{-}trans}$ and PC-MBC$_{mixture}$ 12

3.1 NMR spectra of PC-MBC$_{cis\text{-}cis}$, PC-MBC$_{cis\text{-}trans}$, PC-MBC$_{trans\text{-}trans}$ and PC-MBC$_{mixture}$ 12

3.2 TGA plots of PC-MBC$_{cis\text{-}cis}$, PC-MBC$_{cis\text{-}trans}$, PC-MBC$_{trans\text{-}trans}$ and PC-MBC$_{mixture}$ 20

3.3 DSC traces of PC-MBC$_{cis\text{-}cis}$, PC-MBC$_{cis\text{-}trans}$, PC-MBC$_{trans\text{-}trans}$ and PC-MBC$_{mixture}$ 22

3.4 GPC traces of PC-MBC$_{cis\text{-}cis}$, PC-MBC$_{cis\text{-}trans}$, PC-MBC$_{trans\text{-}trans}$ and PC-MBC$_{mixture}$ 24

Supplementary references 28

Supplementary Note 1. General information

Column chromatography was performed using Merck silica gel type 9385 230–400 mesh and typically dichloromethane and methanol or EtOAc and pentane as eluent.

Thin layer chromatography (TLC): Merck silica gel 60, 0.25 mm. The individual components were visualized by UV or $KMnO_4$ staining.

Gas Chromatography (GC) was used for product identification as well as the determination of the conversion and selectivity values.

Gel Permeation Chromatography (GPC) was conducted at the Graz University of Technology on a Shimadzu instrument equipped with two separating columns from MZ-Gel SD plus (8×300 mm, 5µm) plus 1×precolumn (8×50mm, 5µm). The columns were operated at ambient temperature with a flow-rate of 1 $mL \cdot min^{-1}$ of chloroform. Detection was accomplished at ambient temperature using a RID-20A Differential Refractive Index Detector in series. The molecular weight determination was performed using polystyrene standards of known molecular weight distribution.

Nuclear Magnetic Resonance (NMR) spectroscopy: ^1H, and ^{13}C NMR spectra were recorded on a Bruker Avance III 300 MHz (300 and 75 MHz, respectively) and 2D NMR spectra were recorded on a Bruker Avance III 700 MHz equipped from a 5mm Triple-Resonance cryoprobe (700 and 175 MHz, respectively). All NMR spectra were recorded at RT. Chemical shift values are reported in ppm with the solvent resonance as the internal standard ($CDCl_3$: 7.26 for ^1H, 77.0 for ^{13}C; CD_3OD: 3.31 for ^1H, 49.0 for ^{13}C; DMSO-d_6: 2.50 for ^1H, 39.5 for ^{13}C). Data are reported as follows: chemical shifts, multiplicity (s = singlet, d = doublet, t = triplet, q = quartet, br. = broad, m = multiplet), coupling constants (Hz), and integration.

Differential Scanning Calorimetry (DSC) was conducted at the Graz University of Technology on a Perkin Elmer DSC 8500. In a typical procedure, the sample (5-10 mg) was weighed into a DSC aluminum pan and then capped with a lid. The sample was sealed and heated from 25 to 250 °C with a heating rate of 20 $°C \cdot min^{-1}$. Then it was cooled to 25 °C with a heating rate of 20 $°C \cdot min^{-1}$. Subsequently, a second heating scan to 250 °C with the same heating rate was performed. All of the experiments were performed under an N_2 flow with a flow rate of 20 $mL \cdot min^{-1}$.

Thermogravimetric analysis (TGA) was performed at the Graz University of Technology on a Netzsch Jupiter STA 449C thermogravimetric analyzer. Typically, the sample (1-3 mg) was weighed into a platinum pan. The sample was heated from 20 to 550 °C with a heating rate of 10 $°C \cdot min^{-1}$ and a N_2 flow rate of 20 $mL \cdot min^{-1}$. The temperatures were recorded when 5 % weight loss ($T_{5\%}$) and 90% weight loss rate ($T_{90\,\%}$) occurred.

X-ray diffraction (XRD) was performed at the Graz University of Technology on a RIGAKU Miniflex 600 with D/Tex Ultra detector with a CuKα radiation (λ=1.5418 Å). XRD patterns were collected in reflection geometry in the 2-theta range between 0° and 30°.

Attenuated total reflection-Fourier-transform infrared spectroscopy (FTIR) were performed at the University of Groningen using a VERTEX 70 spectrometer in the wave number range of 400-4000 cm^{-1} with a resolution of 4 cm^{-1}, equipped with an ATR geometry.

1.1 Materials and reagents

Diphenyl carbonate (DPC) (> 99.0%) were purchased from TCI chemicals company and Titanium (IV) butoxide (TBT) (> 97.0%) were purchased from Sigma-Aldrich.

1.2 Preparation of the model compounds

Preparation of the MBC diol: The synthesis of 4,4-methylenebiscyclohexanol (MBC) was carried out according to a previously reported procedure.[1] In a typical procedure, a 1L high pressure Parr autoclave was charged with 2 g Raney nickel catalyst, 5 g bisphenol F, 100 mL isopropanol, and equipped with mechanical stirring. The reactor was sealed, purged 3 times with H_2 and then pressurized with H_2 (40 bar). The reactor was heated to 150 °C for 4 h under stirring at 400 rpm. After completion of the reaction, the reactor was cooled to RT. Then 0.1 mL solution was collected through a syringe and injected to GC-MS or GC-FID after filtration through a PTFE filter (0.45 µm). The Raney nickel was separated from the solution by centrifugation and subsequent decantation and additionally washed with isopropanol (3×30 mL). Then the isopropanol soluble fractions were combined and the solvent was removed under reduced pressure. The product 4, 4'-methylenebiscyclohexanol was obtained as a white solid (5.194 g, 24.5 mmol) in a 98% yield as a mixture of isomers (cis-cis: cis-trans: trans: trans with the ratio of 10: 43: 47), based on ^{1}H NMR.

Purification protocols for separation of MBC isomers: In a typical procedure, a 100 mL round bottom flask, equipped with a magnetic stirring bar, was charged with 5 g of $MBC_{cis\text{-}cis:\ cis\text{-}trans:\ trans\text{-}trans}$ and 50 mL chloroform. The mixture was heated to 50 °C under stirring at 500 rpm until all MBC was solubilized. Then, the mixture was allowed to cool in the fridge for two days to yield solid crystals (2.5 g) by filtration. The pre-recrystallized solid crystals were subjected to secondary recrystallization to yield pure $\mathbf{MBC}_{trans\text{-}trans}$ (1.3 g). The $MBC_{cis\text{-}trans}$ and $MBC_{cis\text{-}cis}$ were separated by repeatedly column chromatography on silica gel, using ethyl acetate: hexane (1:3) as eluent.

1.3 Conversion, selectivity and yield calculation

i For copolymerization of MBC with DPC

$$\text{Yield (\%)} = \frac{\text{Mass of the obtained polymers}}{\text{Mass of theoretically obtained polymers}} \times 100\%$$

Abbreviations

MBC: 4,4-methylenebiscyclohexanol

DPC: diphenyl carbonate

1.4 General experimental procedures

The synthesis of renewable polycarbonates from MBC diol and its pure isomers. The two-step melt polymerization was performed using equal molar ratio of the MBC diol or its pure isomers and the co-monomer DPC in the presence of Titanium (IV) butoxide (TBT) as a catalyst. In short, a 100 mL three-neck flask was charged 2.5 mmol MBC, 2.5 mmol DPC and 1 mol % TBT catalyst, equipped with a magnetic stirrer and reflux condenser. The reaction was performed at 190 °C/N_2 for 1 h under nitrogen flow. Then, the reaction temperature was increased to 230 °C and the pressure was slightly reduced to 1 mba using an oil pump for 1 h. After that, the reaction mixture was cooled down to RT and the pressure was returned to atmospheric pressure by the introduction of nitrogen gas. The obtained solid was then dissolved in $CHCl_3$ and subsequently precipitated in method to yield purified polymers which were characterized by NMR, GPC, DSC, TGA, FTIR.

Supplementary Note 2. Analysis and characterizations of MBC and its isomers

2.1 NMR spectra of MBC and its isomers

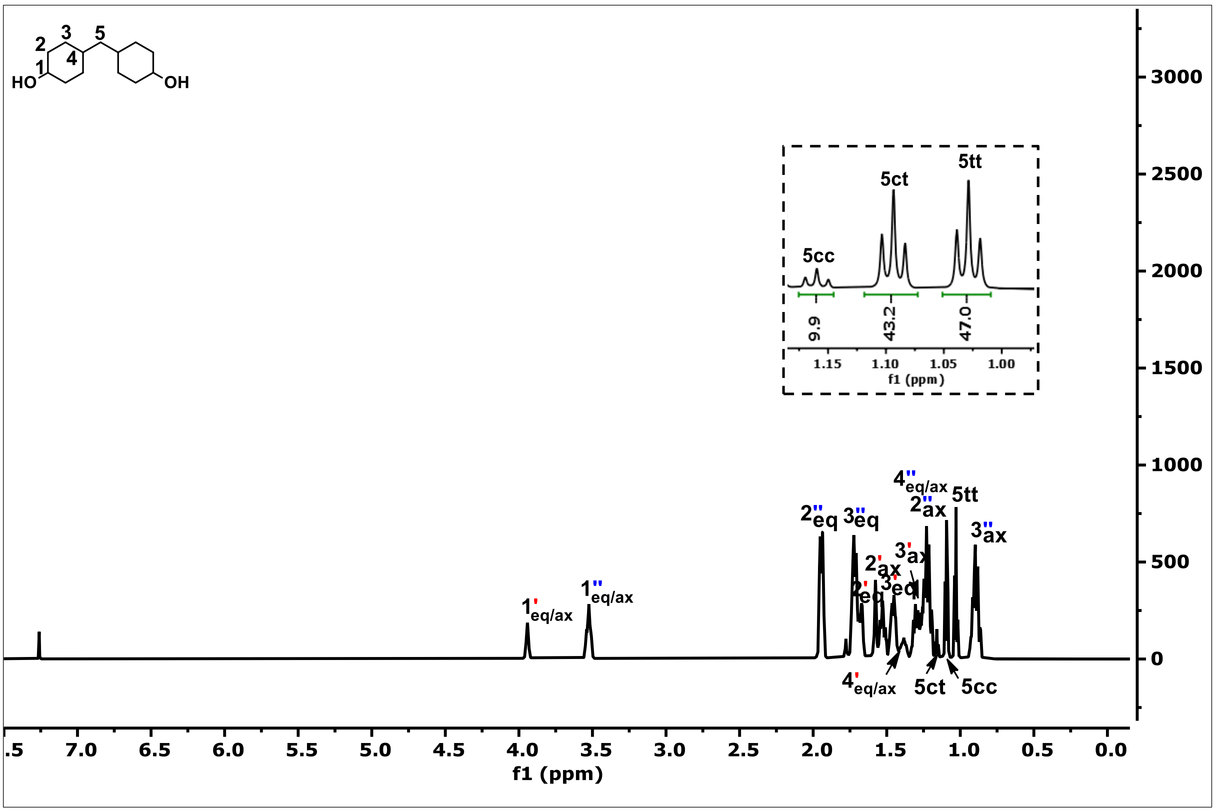

Figure S1 ^{1}H NMR spectrum of $MBC_{cis\text{-}cis,\ cis\text{-}trans,\ trans\text{-}trans}$ ($MBC_{mixture}$)

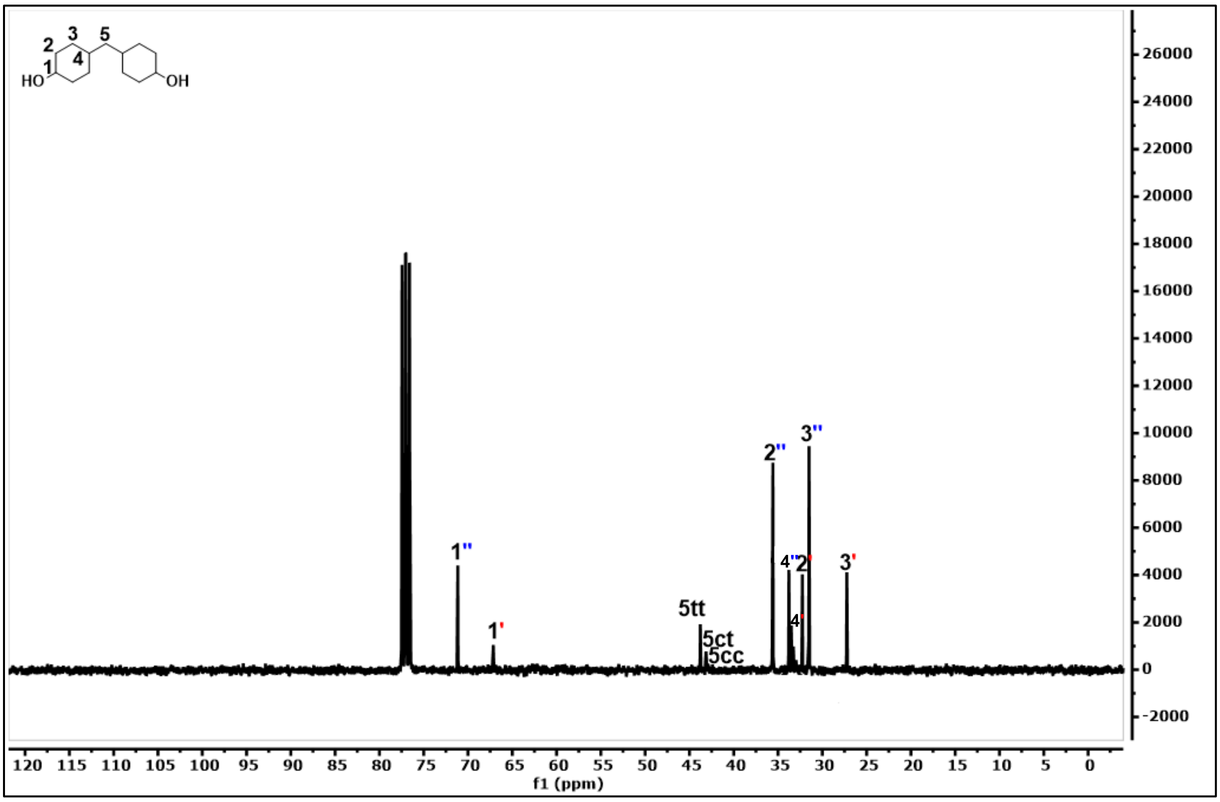

Figure S2 ^{13}C NMR spectrum of $MBC_{cis\text{-}cis,\ cis\text{-}trans,\ trans\text{-}trans}$

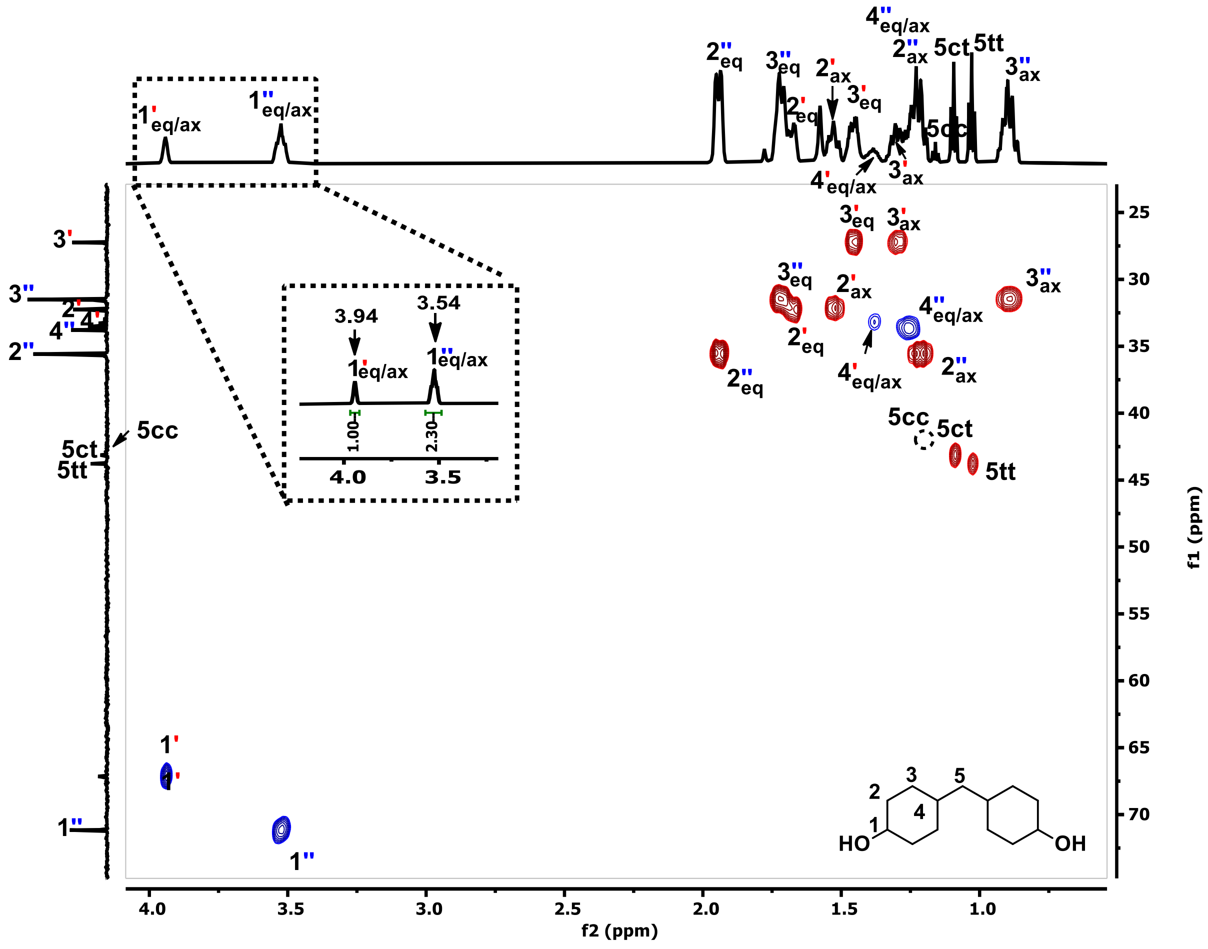

Figure S3 HSQC spectrum of $MBC_{cis\text{-}cis,\ cis\text{-}trans,\ trans\text{-}trans}$

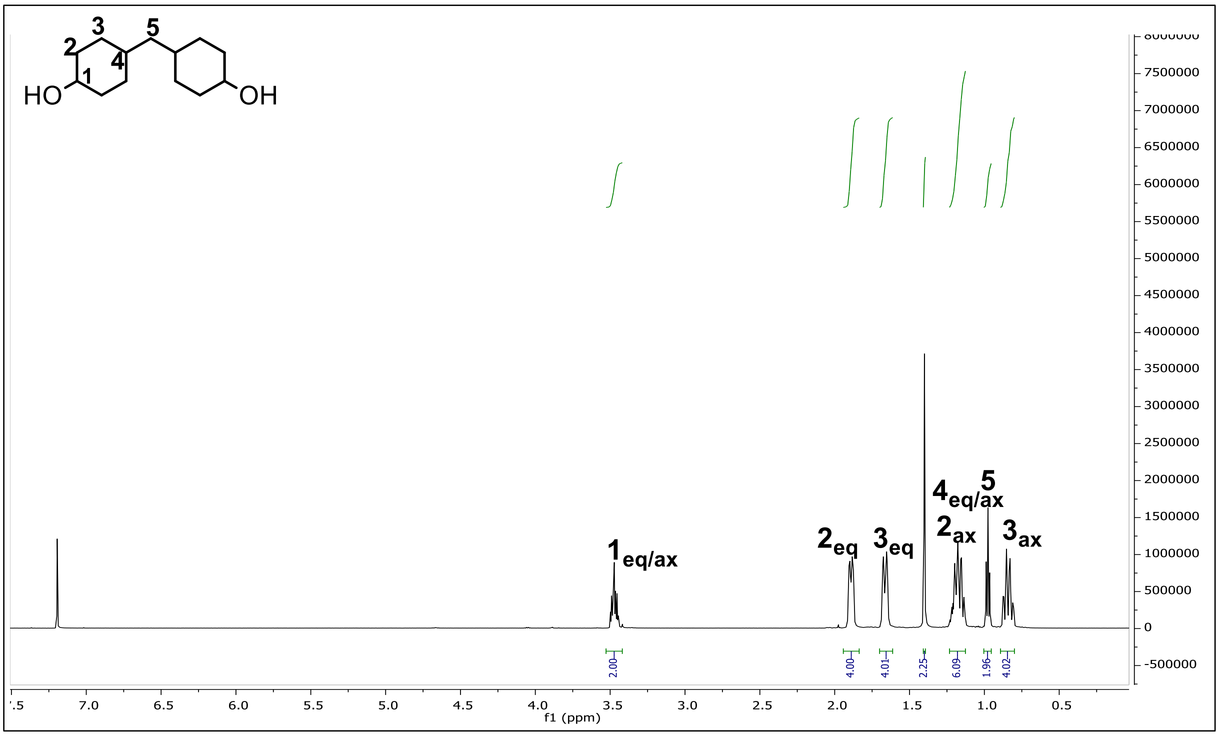

Figure S4 ^{1}H NMR spectrum of $MBC_{trans\text{-}trans}$

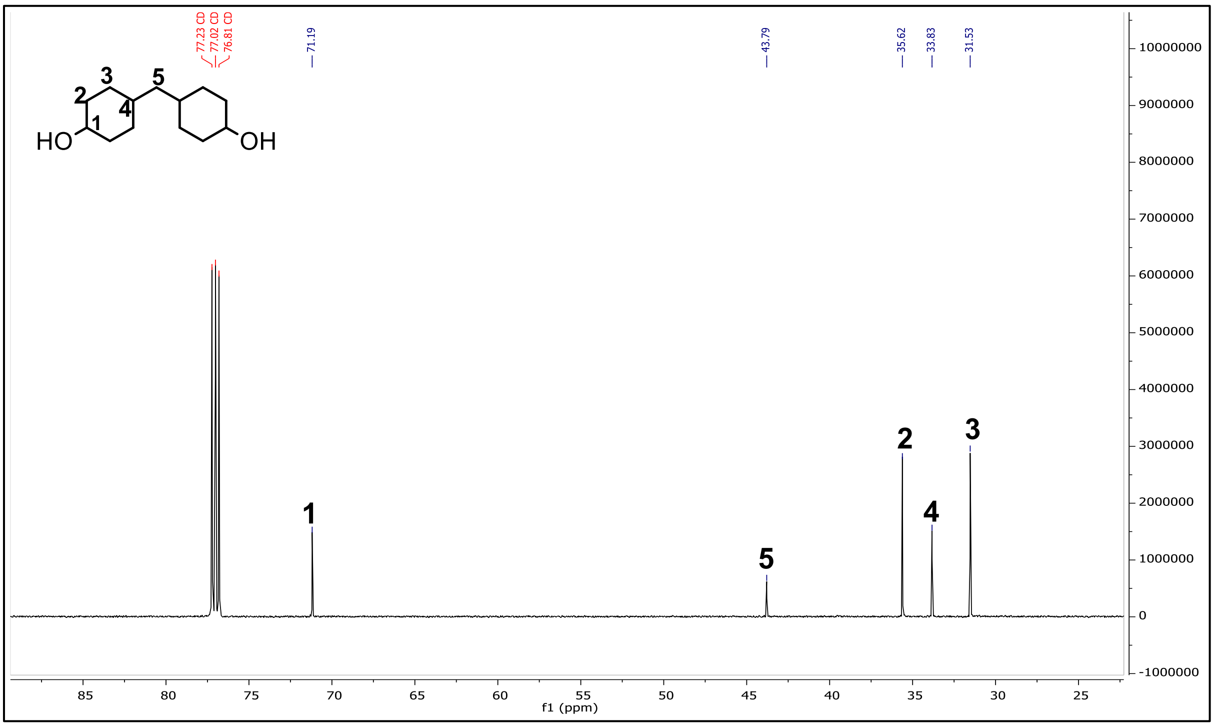

Figure S5 ^{13}C NMR spectrum of MBC$_{\text{trans-trans}}$

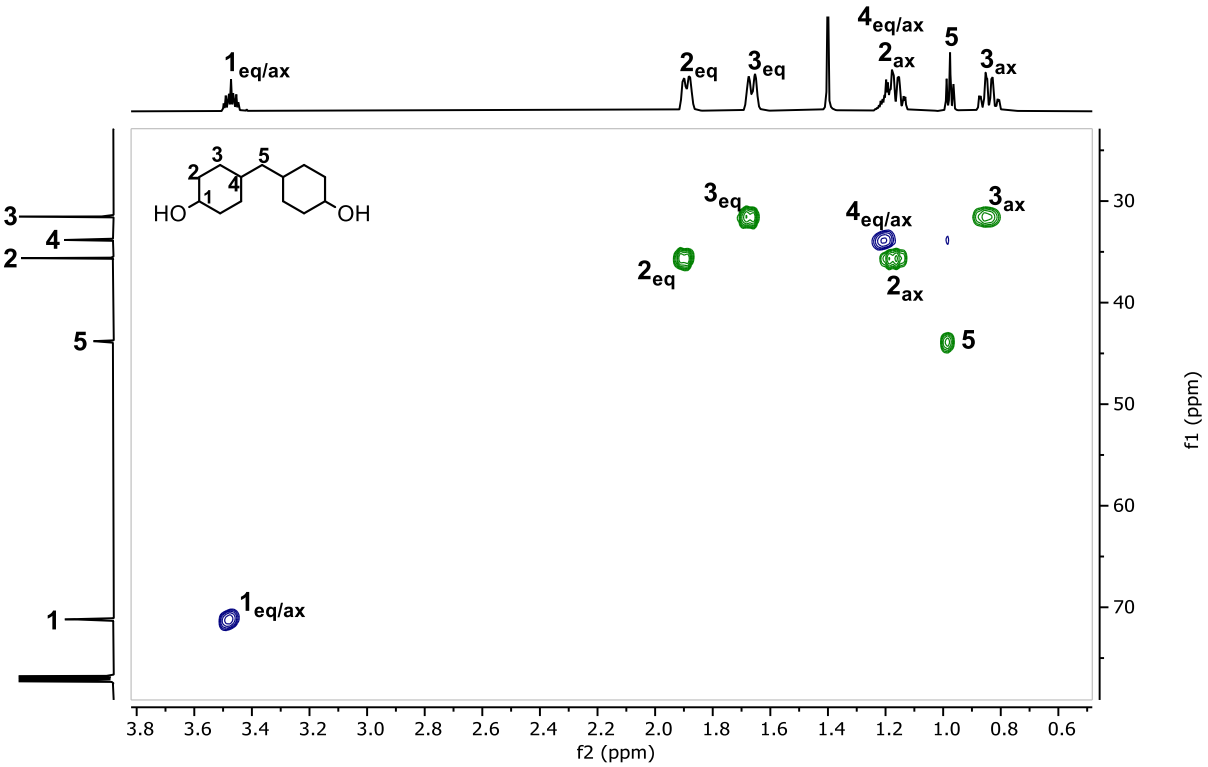

Figure S6 2D HSQC spectrum of MBC$_{\text{trans-trans}}$

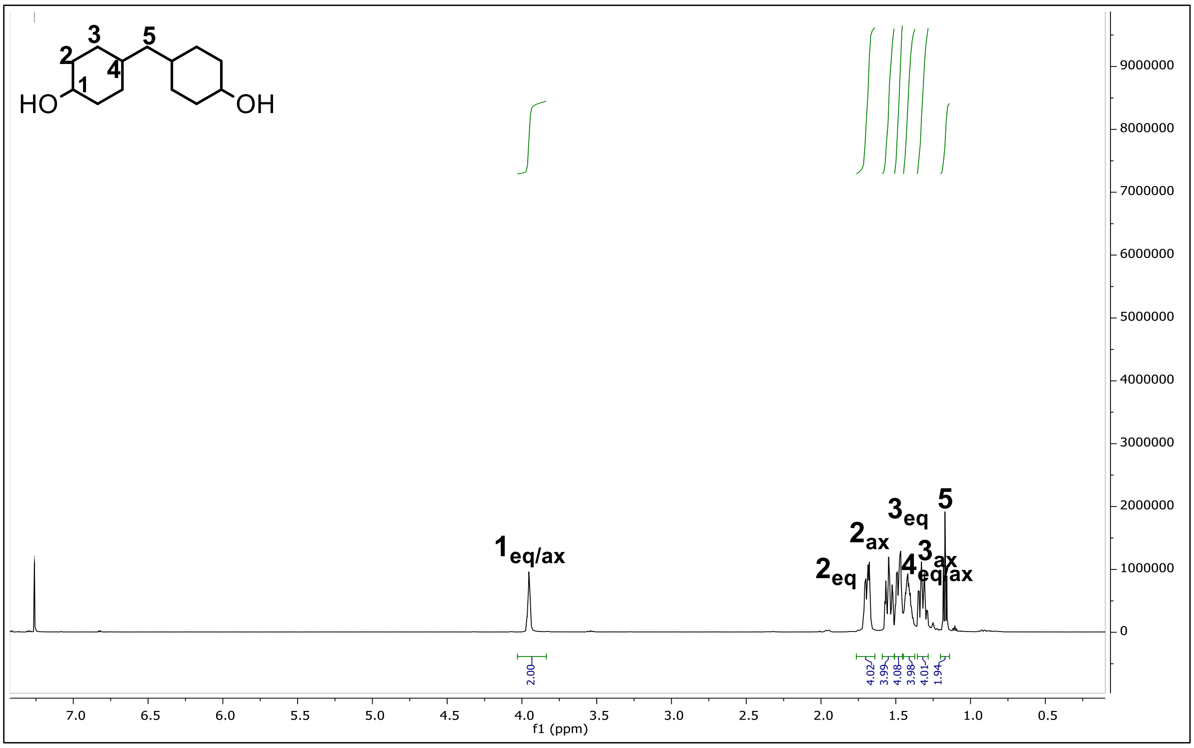

Figure S7 ^{1}H NMR spectrum of MBC$_{cis-cis}$

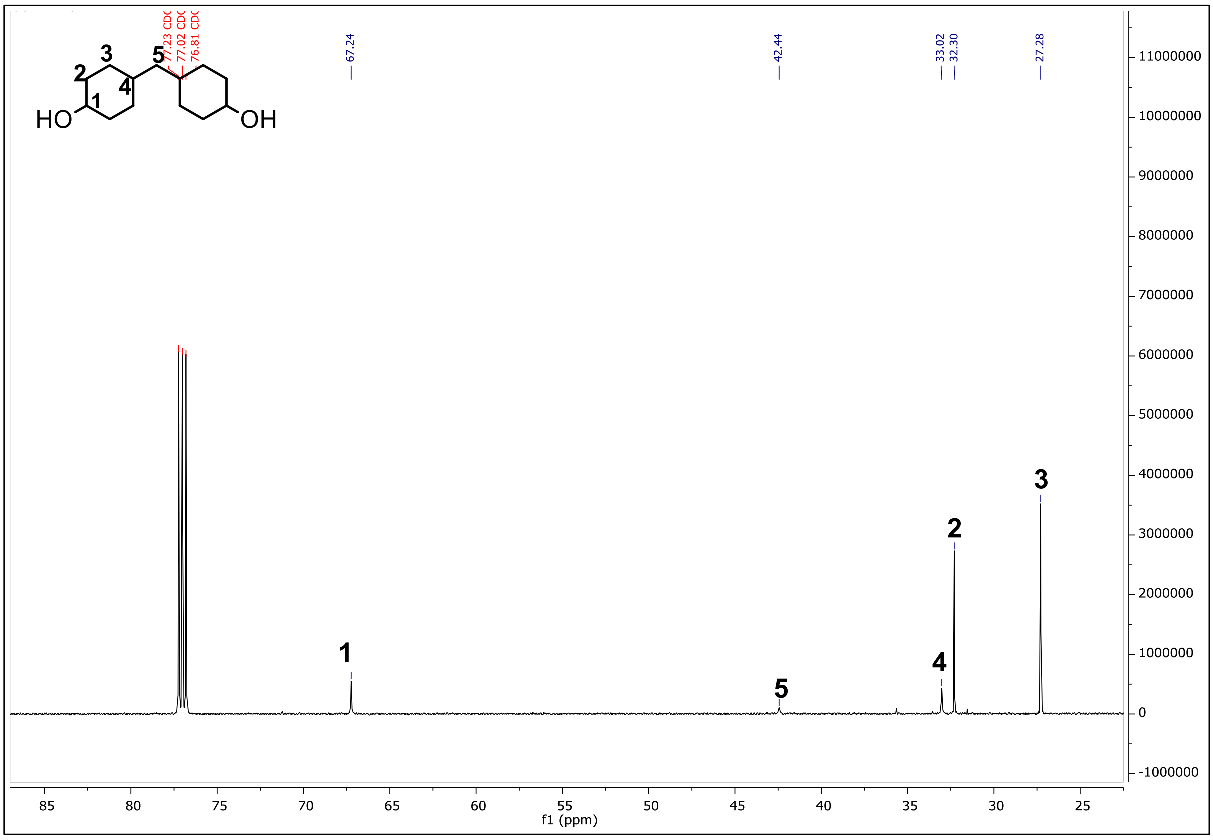

Figure S8 ^{13}C NMR spectrum of MBC$_{cis-cis}$

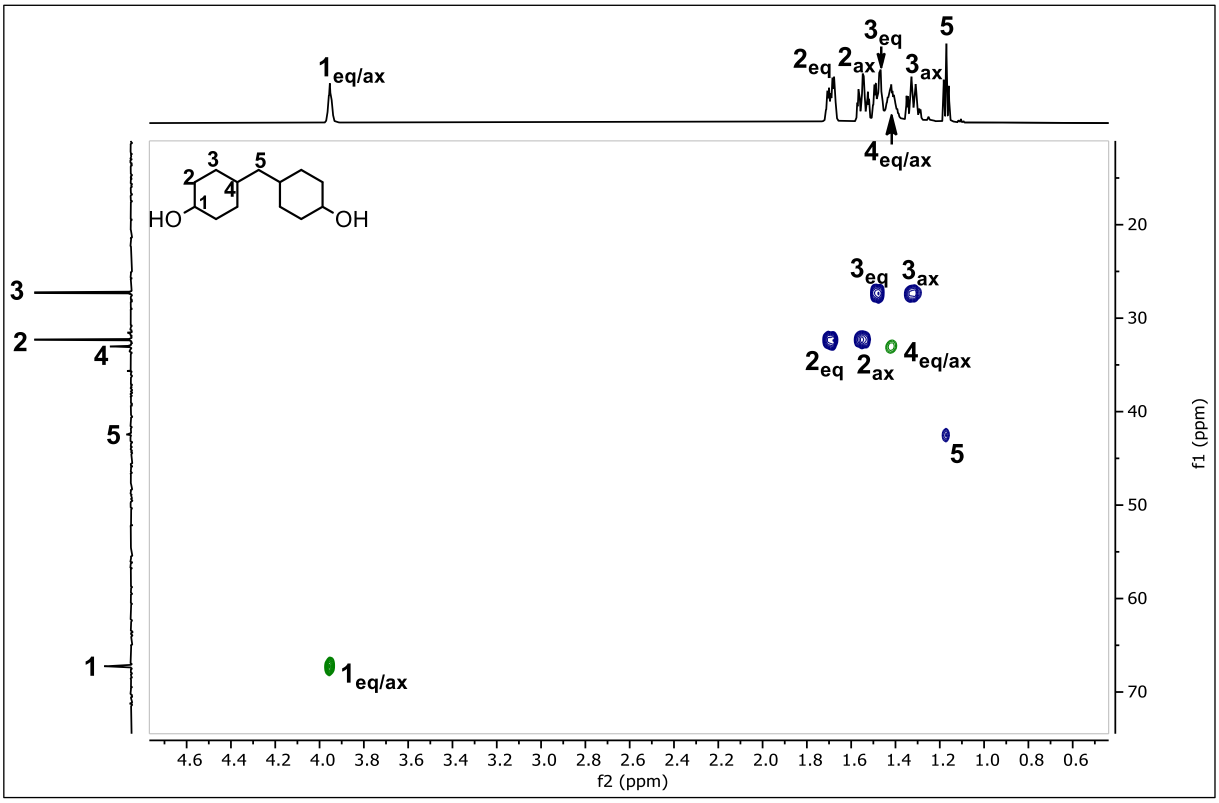

Figure S9 2D HSQC spectrum of $MBC_{cis\text{-}cis}$

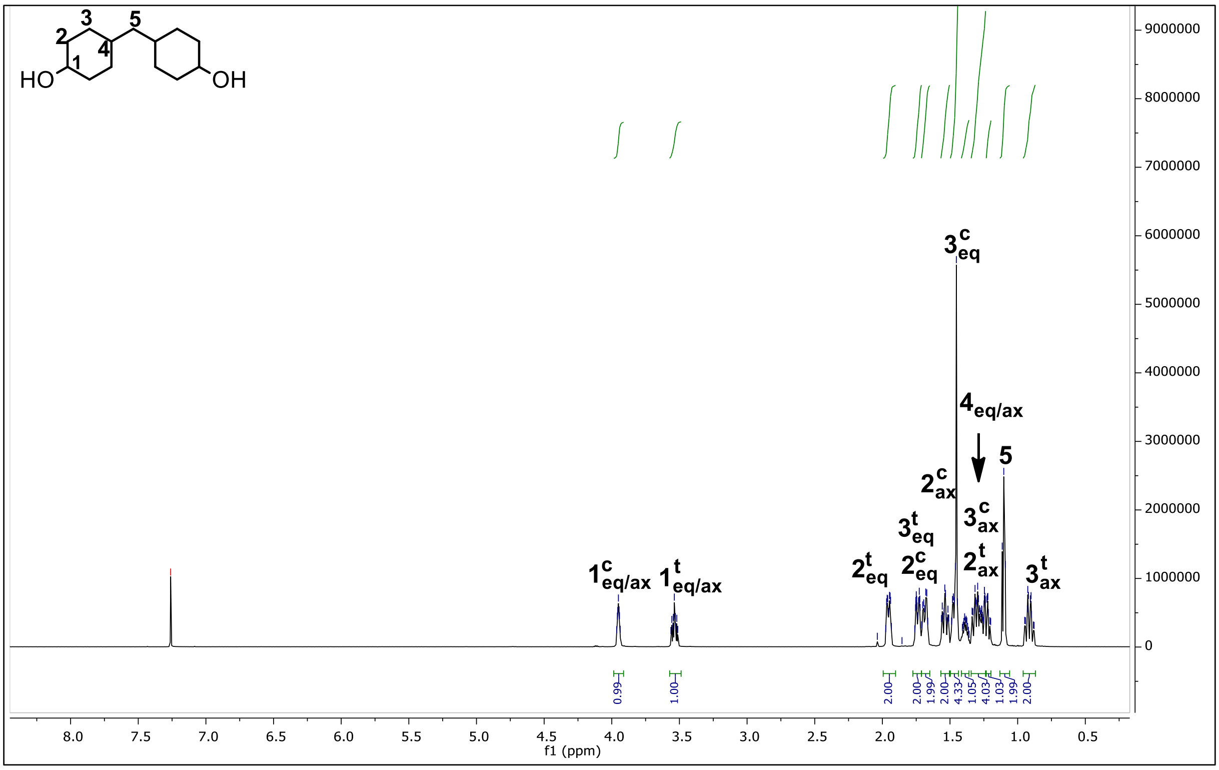

Figure S10 ^{1}H NMR spectrum of $MBC_{cis\text{-}trans}$

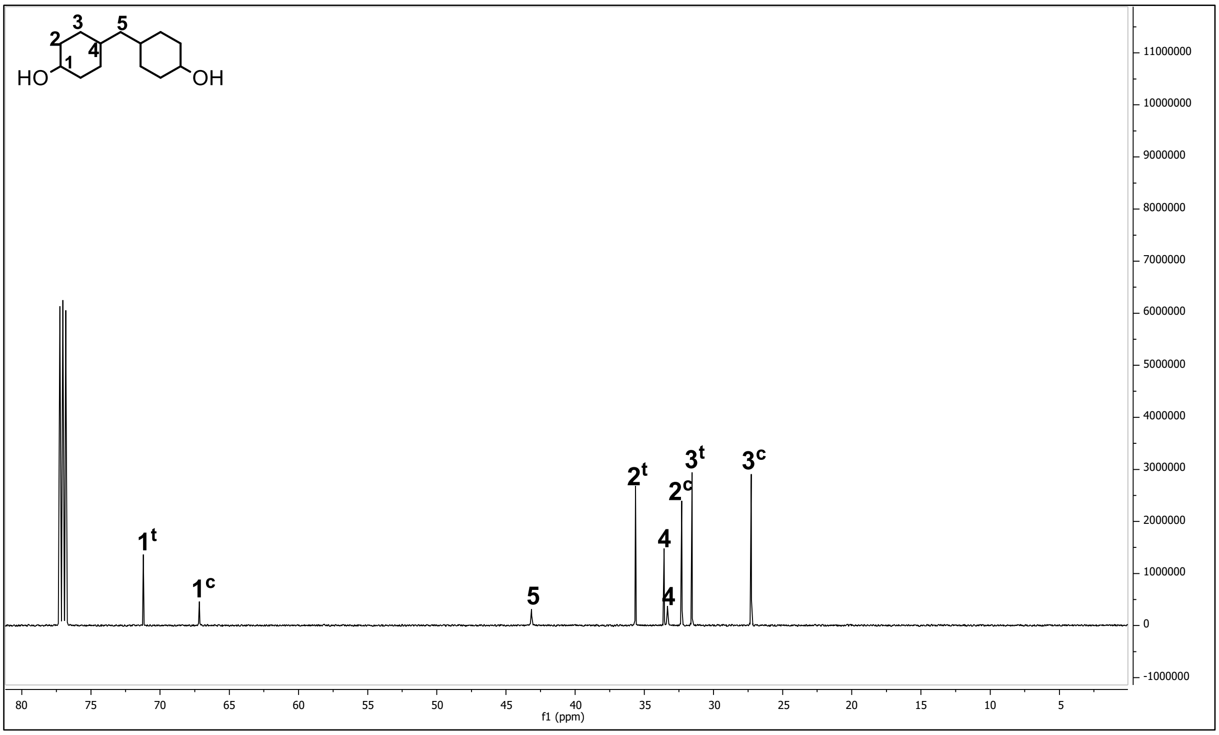

Figure S11 ^{13}C NMR spectrum of $MBC_{cis\text{-}trans}$

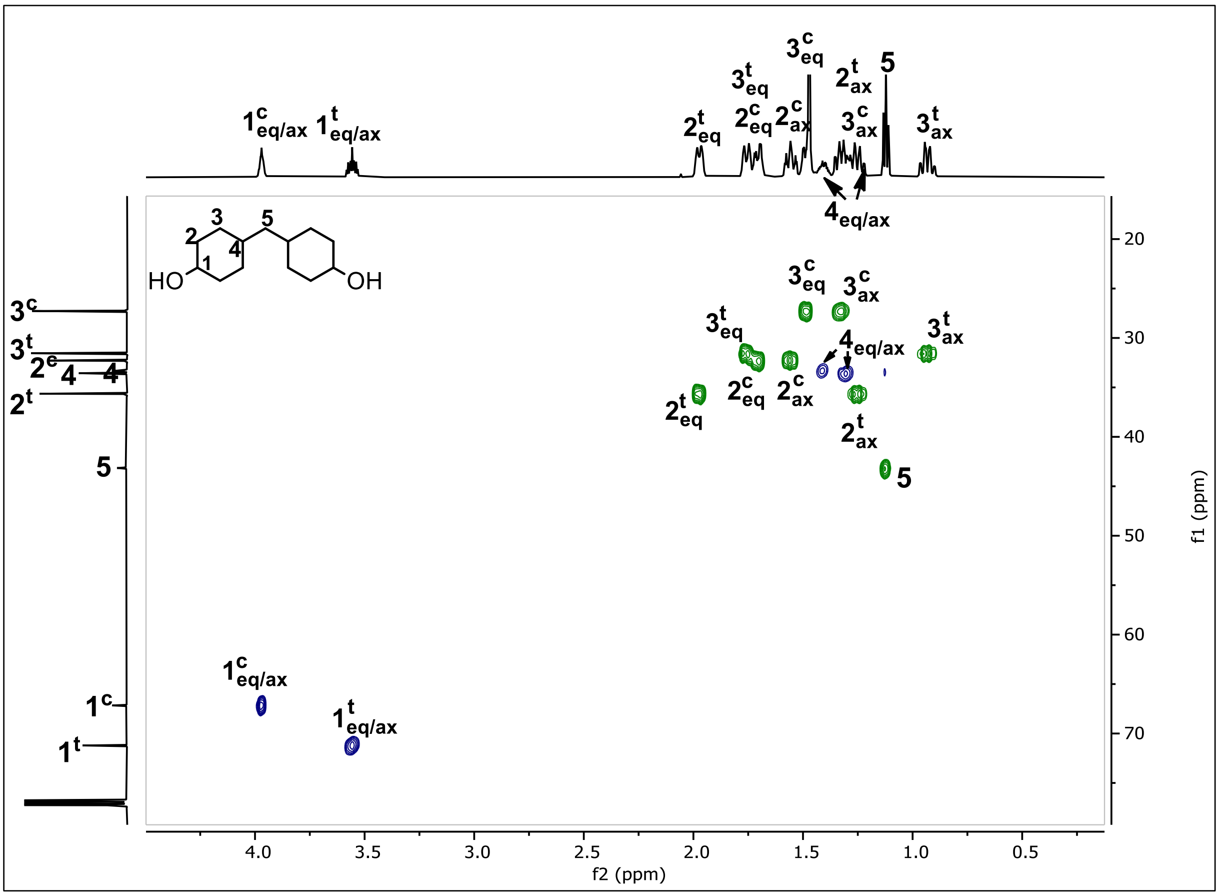

Figure S12 2D HSQC spectrum of $MBC_{cis\text{-}trans}$

2.2 NMR-based structural determination of the cis/trans configurations of the pure MBC isomers

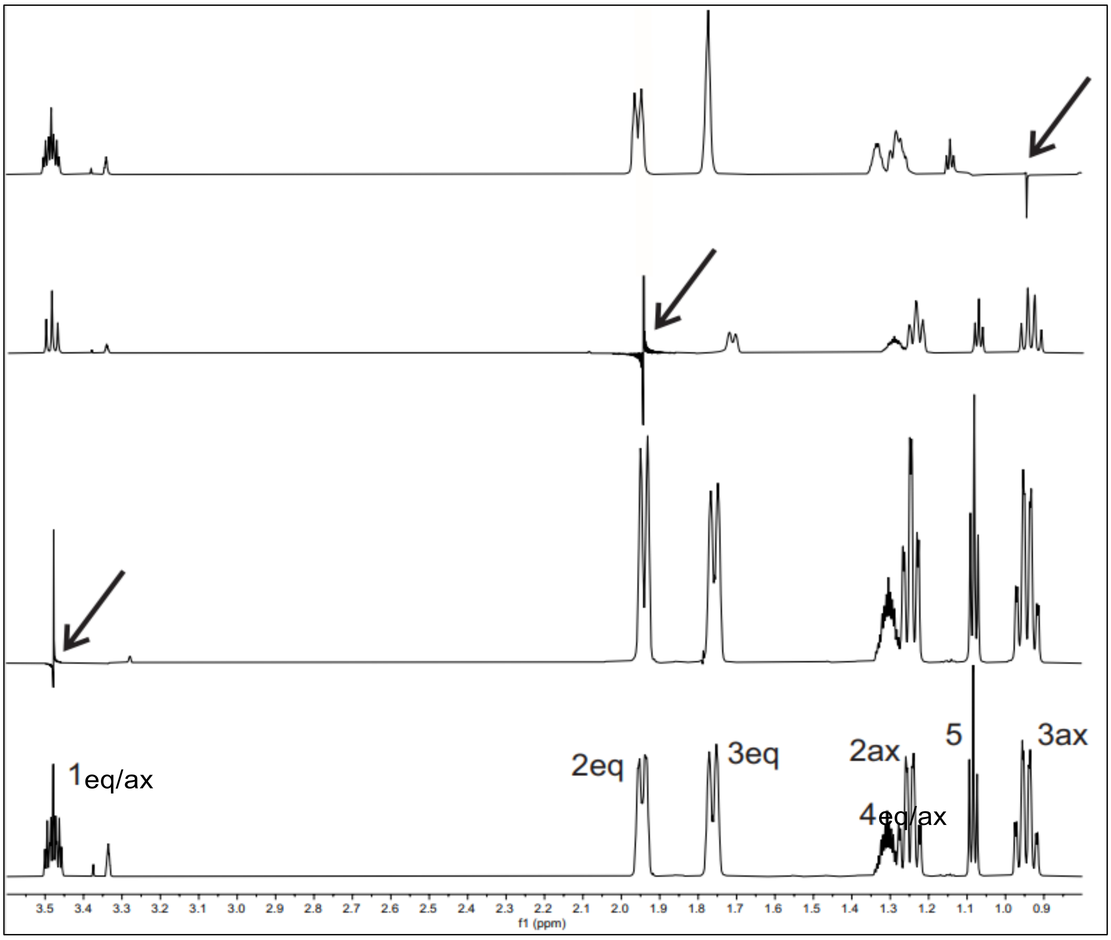

Figure S13 Conventional ^{1}H and various selectively decoupled ^{1}H spectra of $MBC_{trans\text{-}trans}$

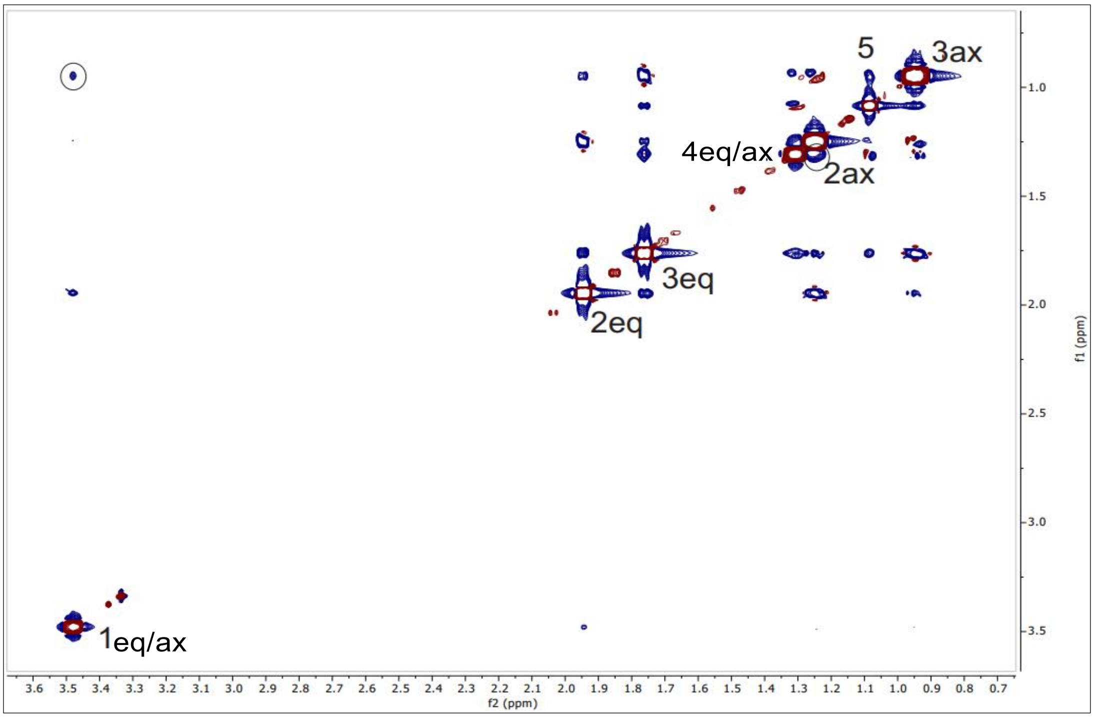

Figure S14 NOESY spectrum of $MBC_{trans\text{-}trans}$ with NOEs indicative of axial Hs circled

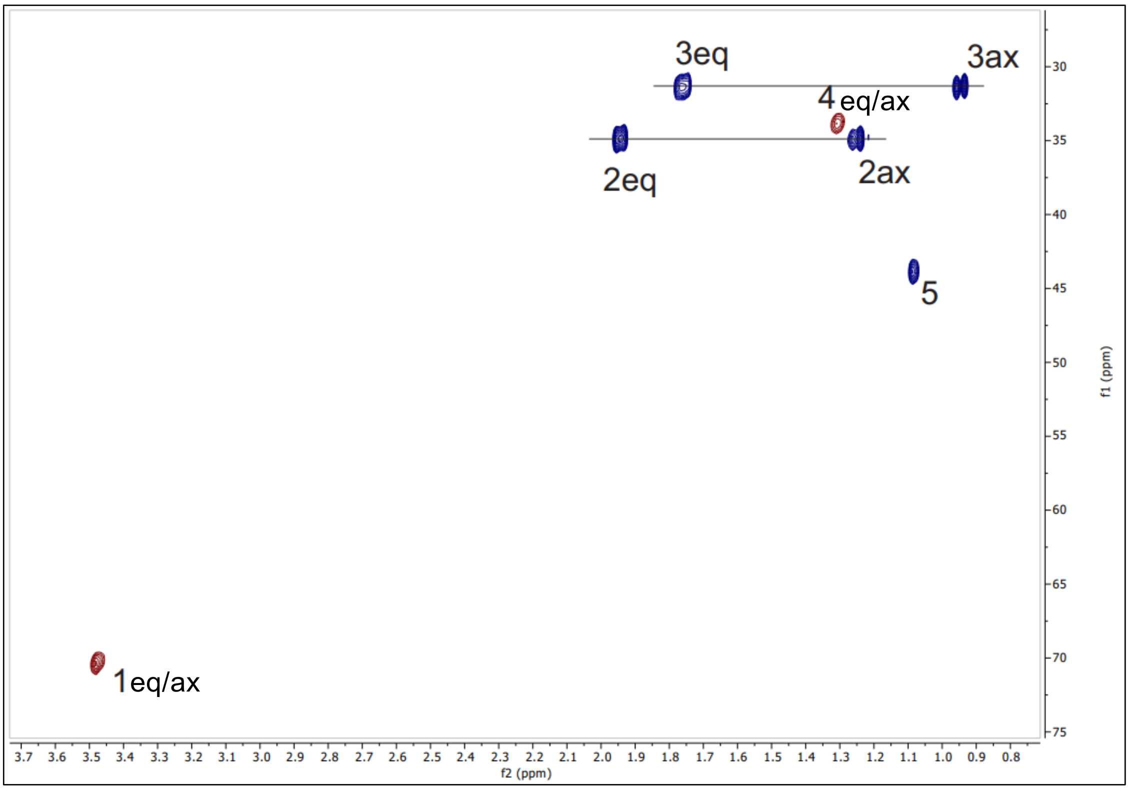

Figure S15 Multiplicity-edited, sensitivity-enhanced ^{1}H-^{13}C HSQC spectra of $MBC_{trans\text{-}trans}$

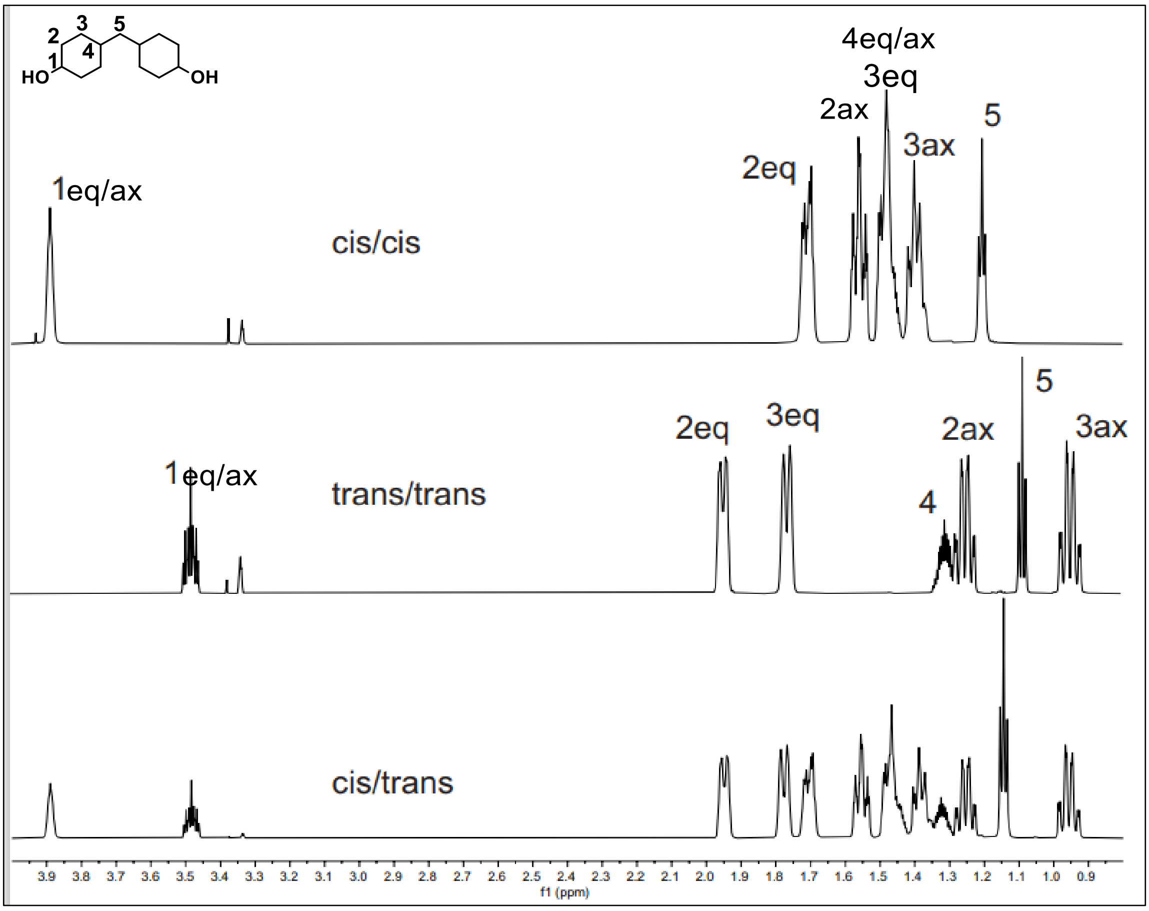

Figure S16 ^{1}H spectra of $MBC_{cis\text{-}cis}$, $MBC_{cis\text{-}trans}$ and $MBC_{trans\text{-}trans}$

Supplementary Note 3. Analysis and characterizations of PC-MBC$_{cis\text{-}cis}$, PC-MBC$_{cis\text{-}trans}$, PC-MBC$_{trans\text{-}trans}$ and PC-MBC$_{mixture}$

3.1 NMR spectra of PC-MBC$_{cis\text{-}cis}$, PC-MBC$_{cis\text{-}trans}$, PC-MBC$_{trans\text{-}trans}$ and PC-MBC$_{mixture}$

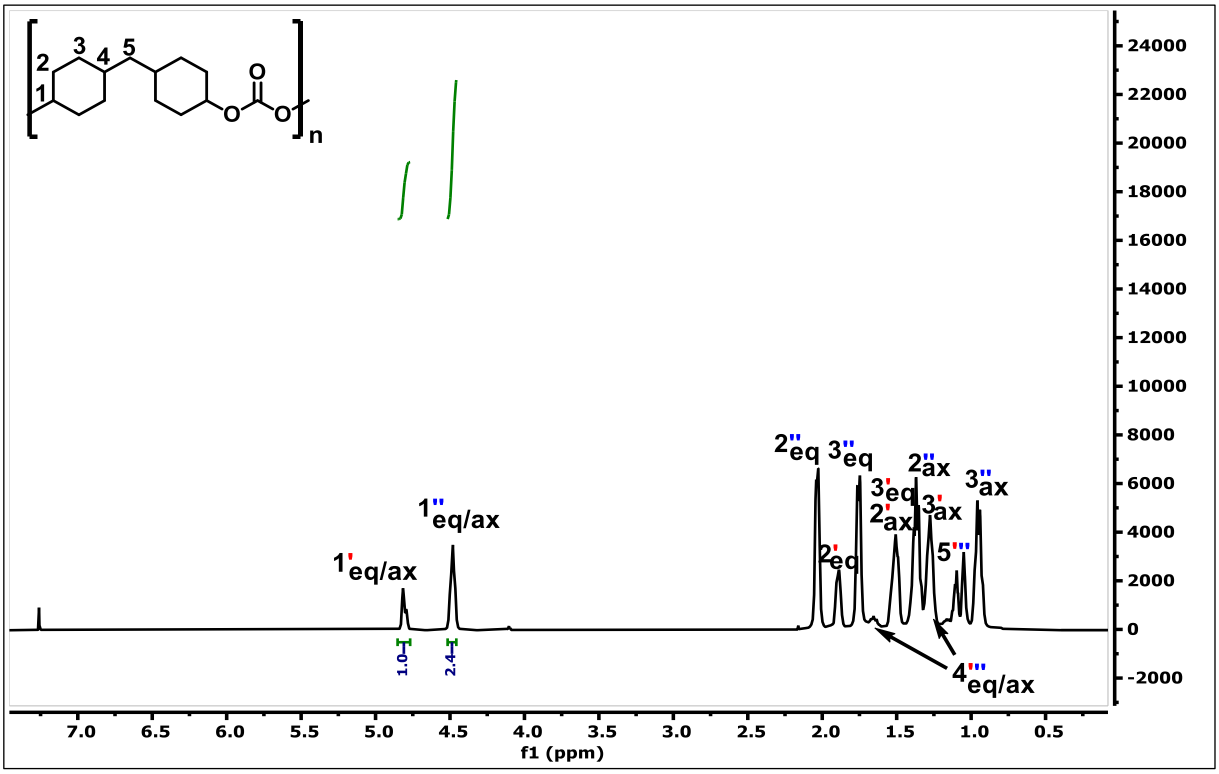

Figure S17 ^{1}H spectrum of PC-MBC$_{mixture}$

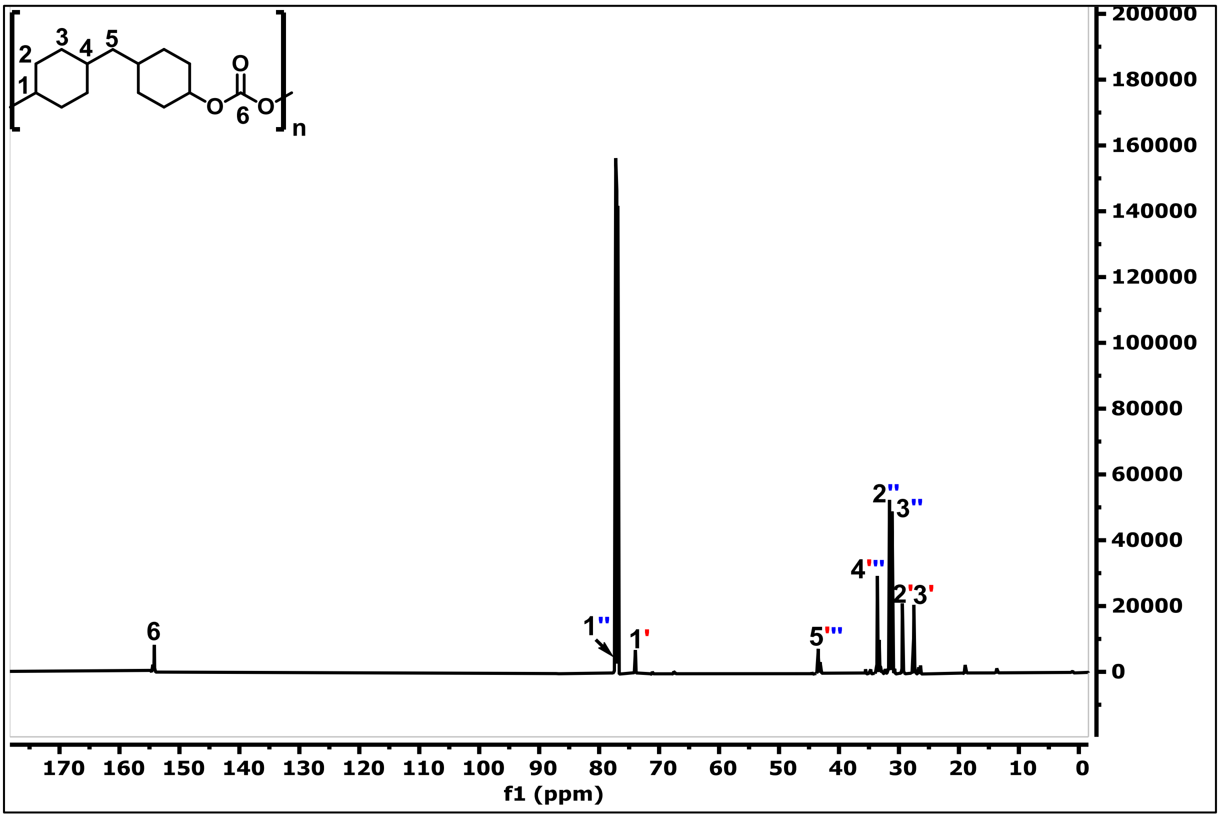

Figure S18 ^{13}C spectrum of PC-MBC$_{mixture}$

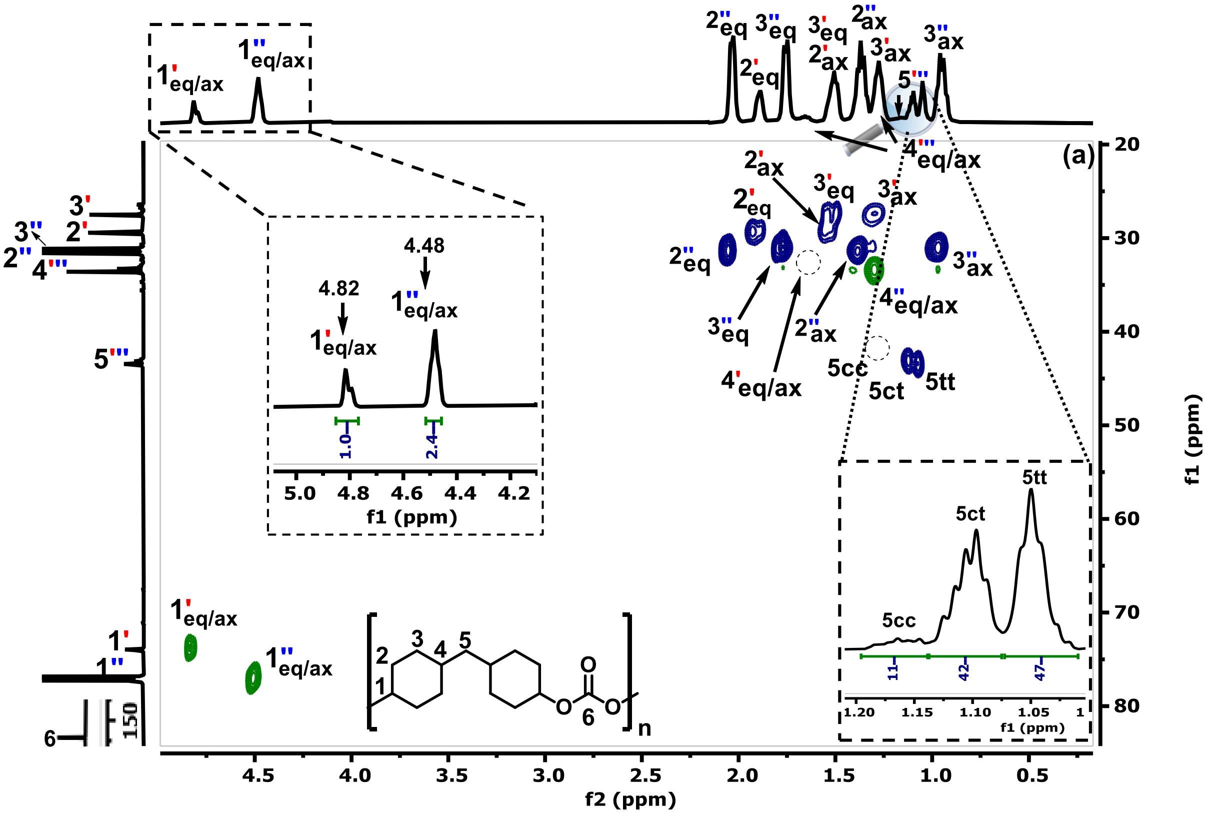

Figure S19 HSQC spectrum of PC-MBC$_{mixture}$

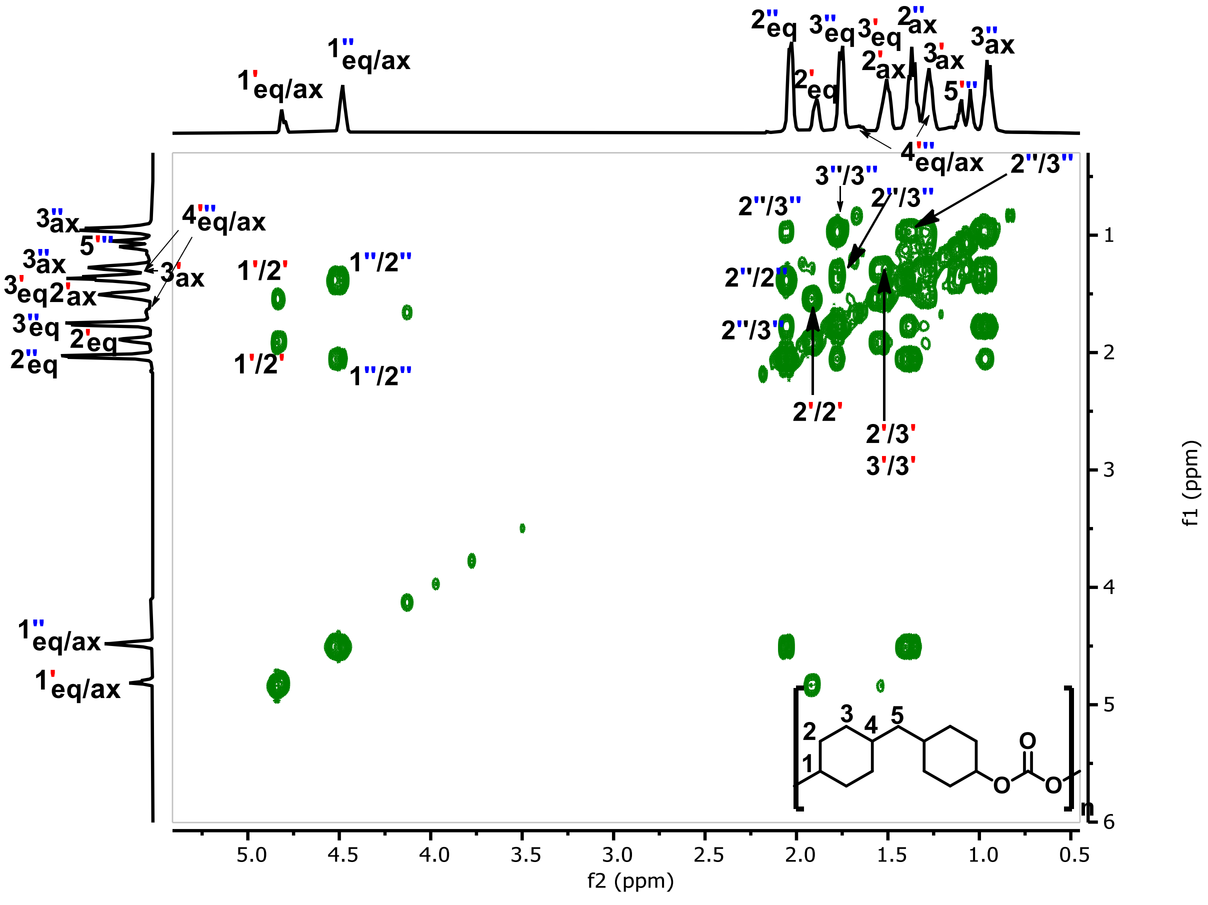

Figure S20 COSY spectrum of PC-MBC$_{mixture}$

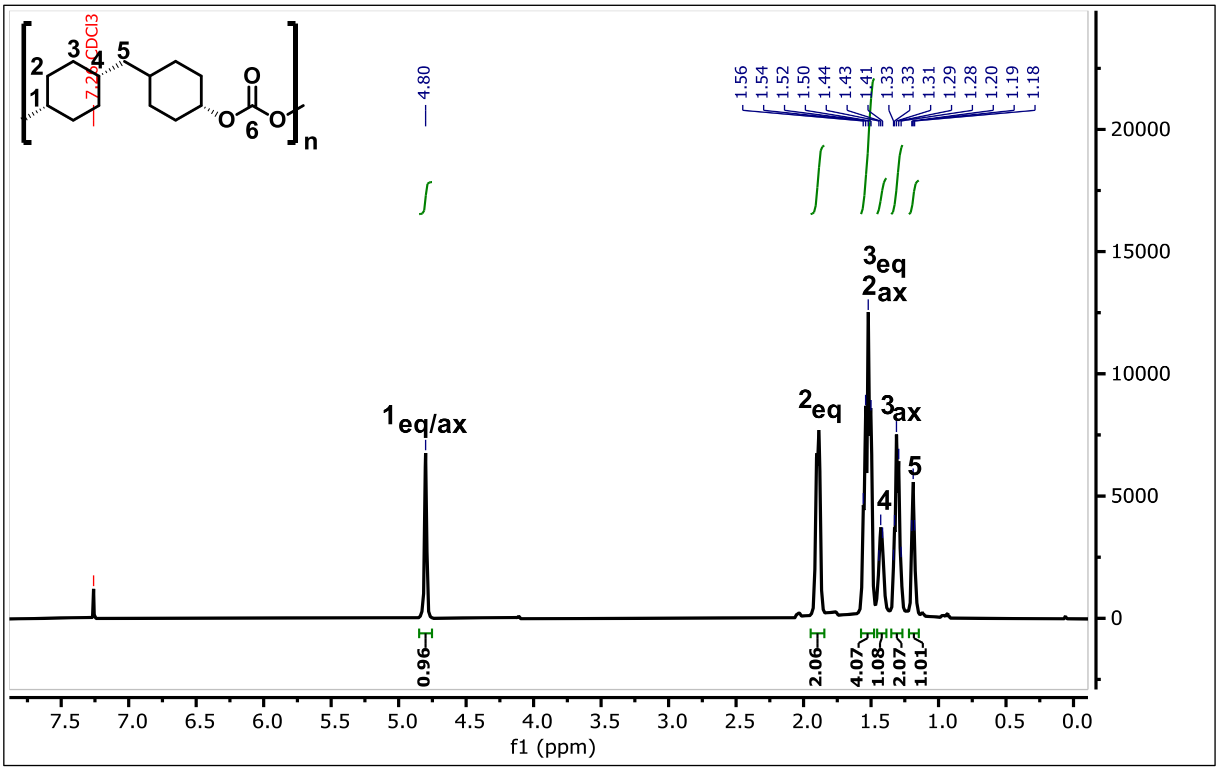

Figure S21 ^{1}H spectrum of PC-MBC$_{cis\text{-}cis}$

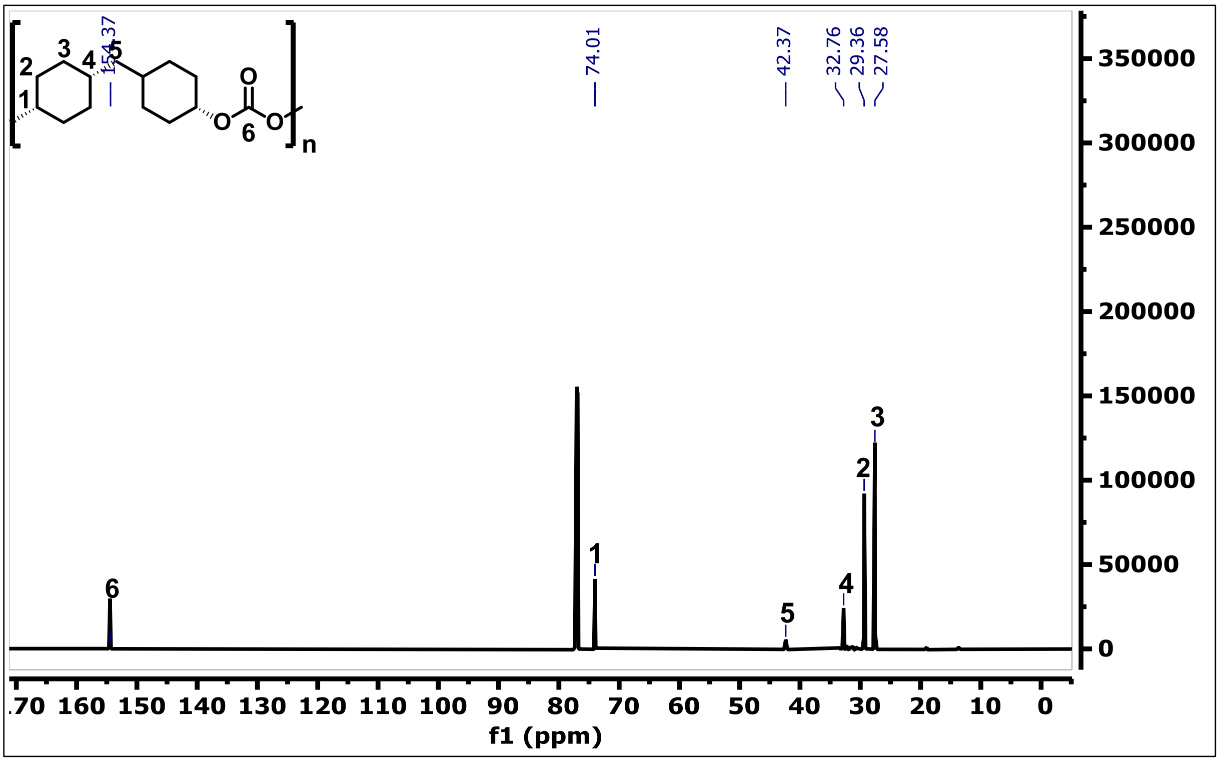

Figure S22 ^{13}C spectrum of PC-MBC$_{cis\text{-}cis}$

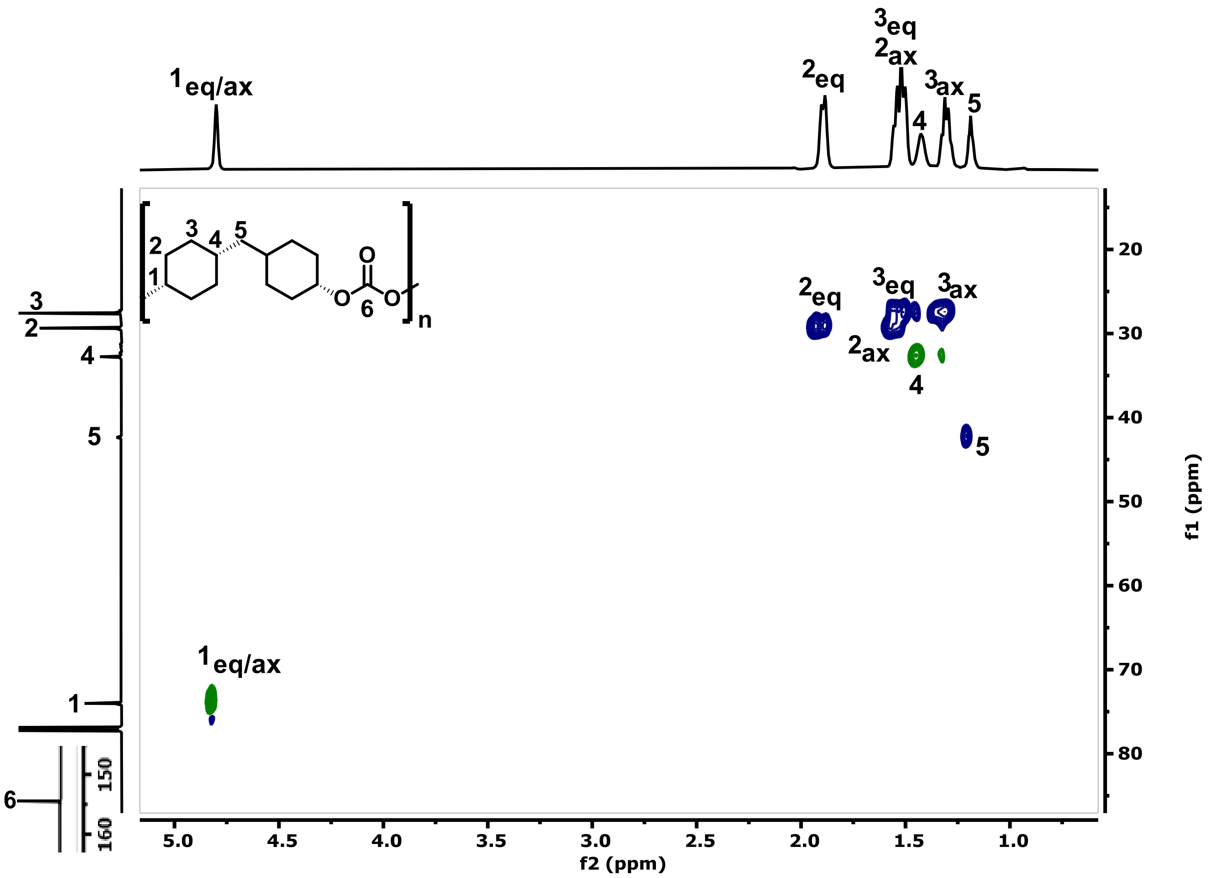

Figure S23 HSQC spectrum of PC-MBC$_{cis\text{-}cis}$

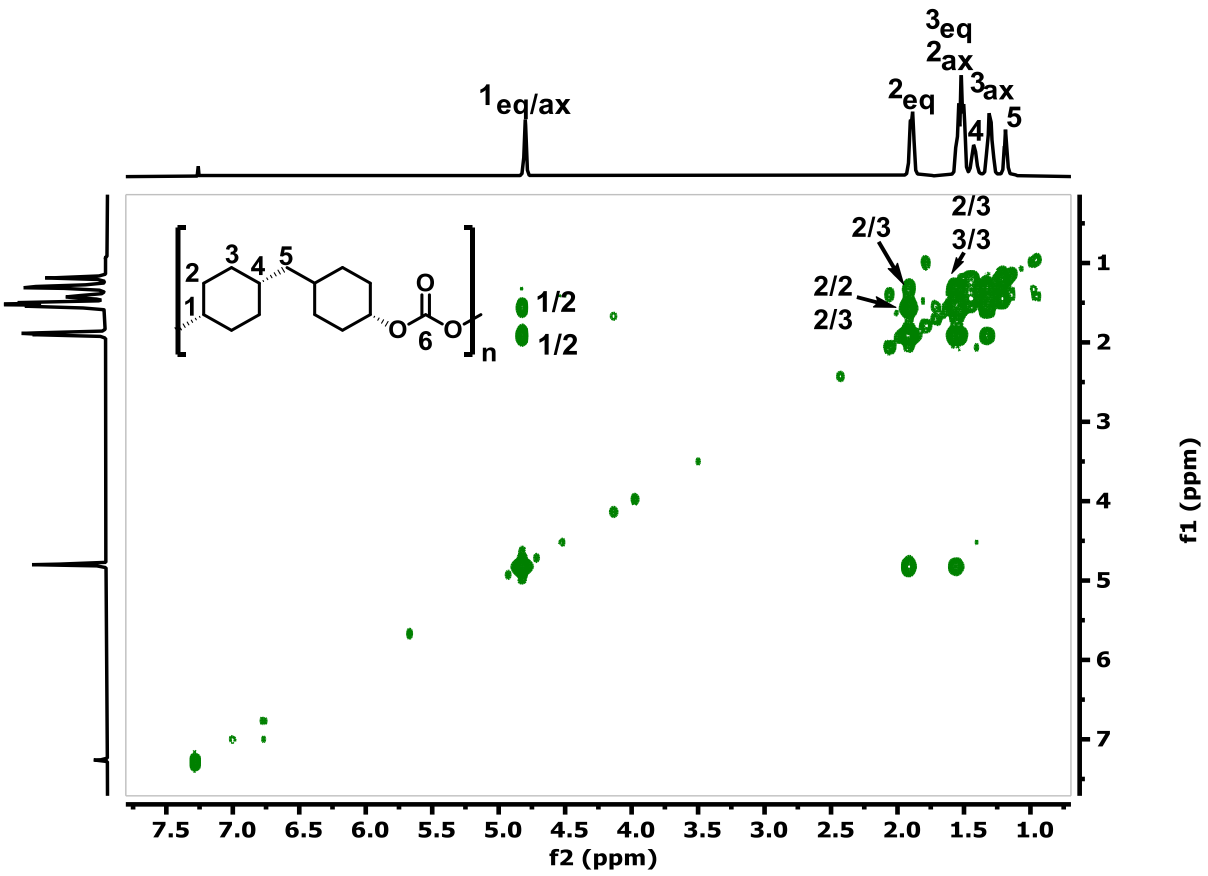

Figure S24 COSY spectrum of PC-MBC$_{cis\text{-}cis}$

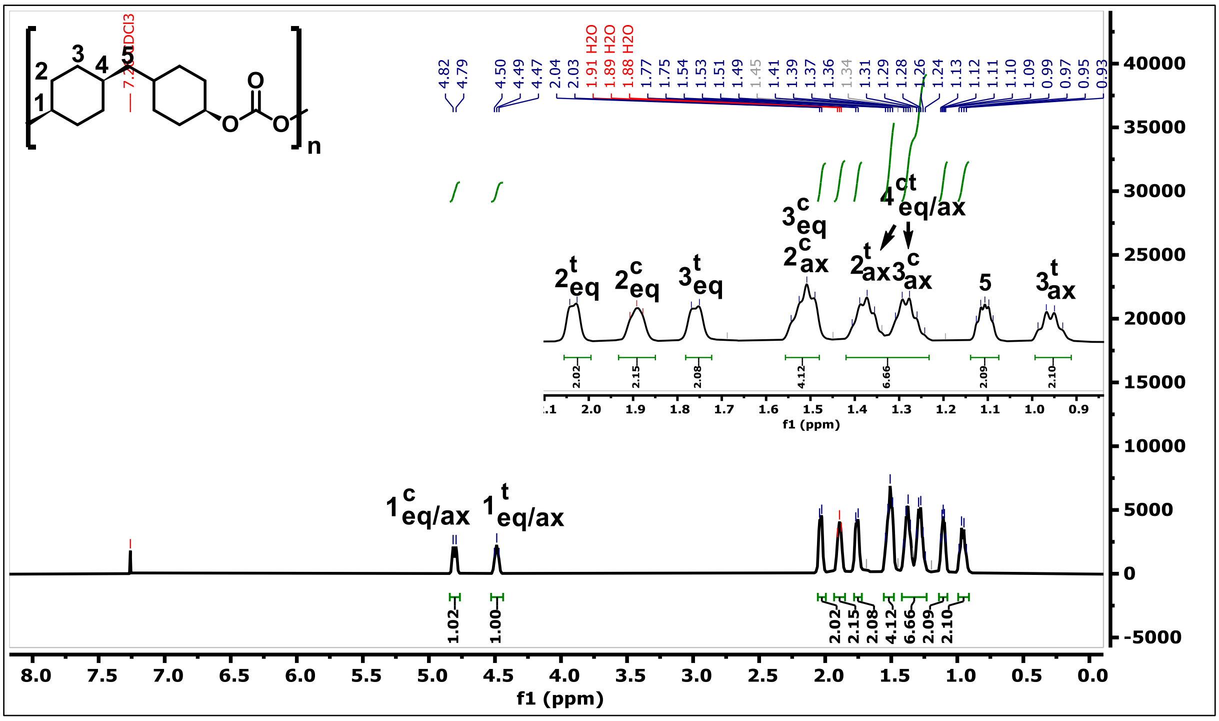

Figure S25^{1}H spectrum of PC-MBC$_{cis\text{-}trans}$

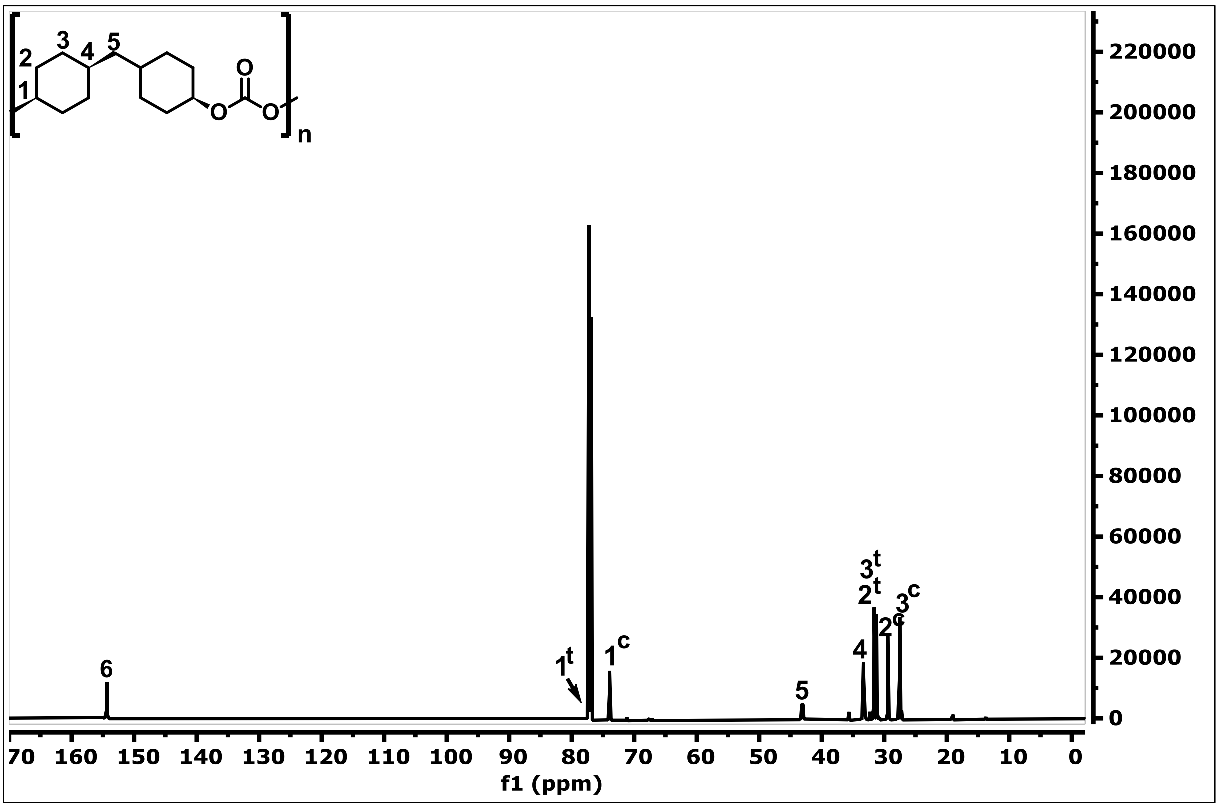

Figure S26 ^{13}C spectrum of PC-MBC$_{cis\text{-}trans}$

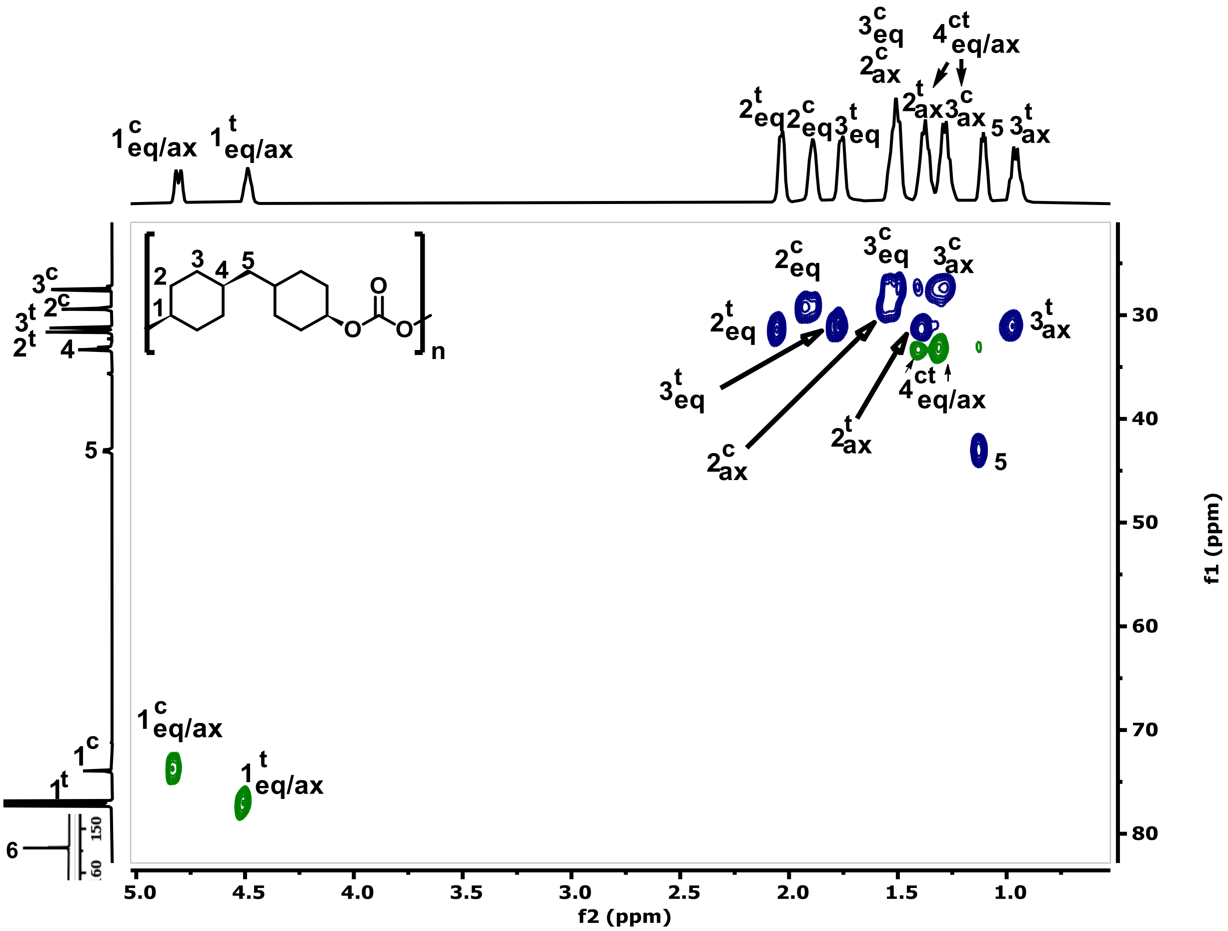

Figure S27 HSQC spectrum of PC-MBC$_{cis\text{-}trans}$

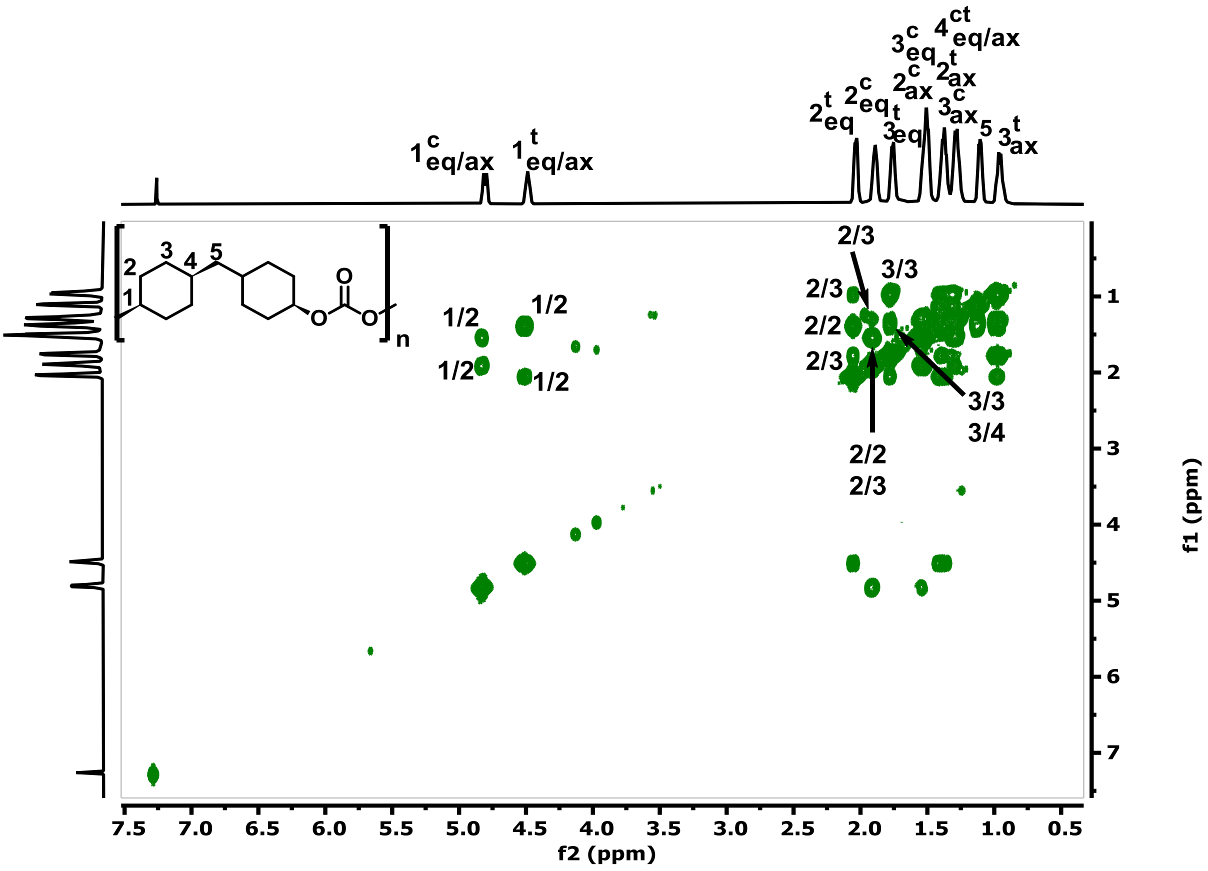

Figure S28 COSY spectrum of PC-MBC$_{cis\text{-}trans}$

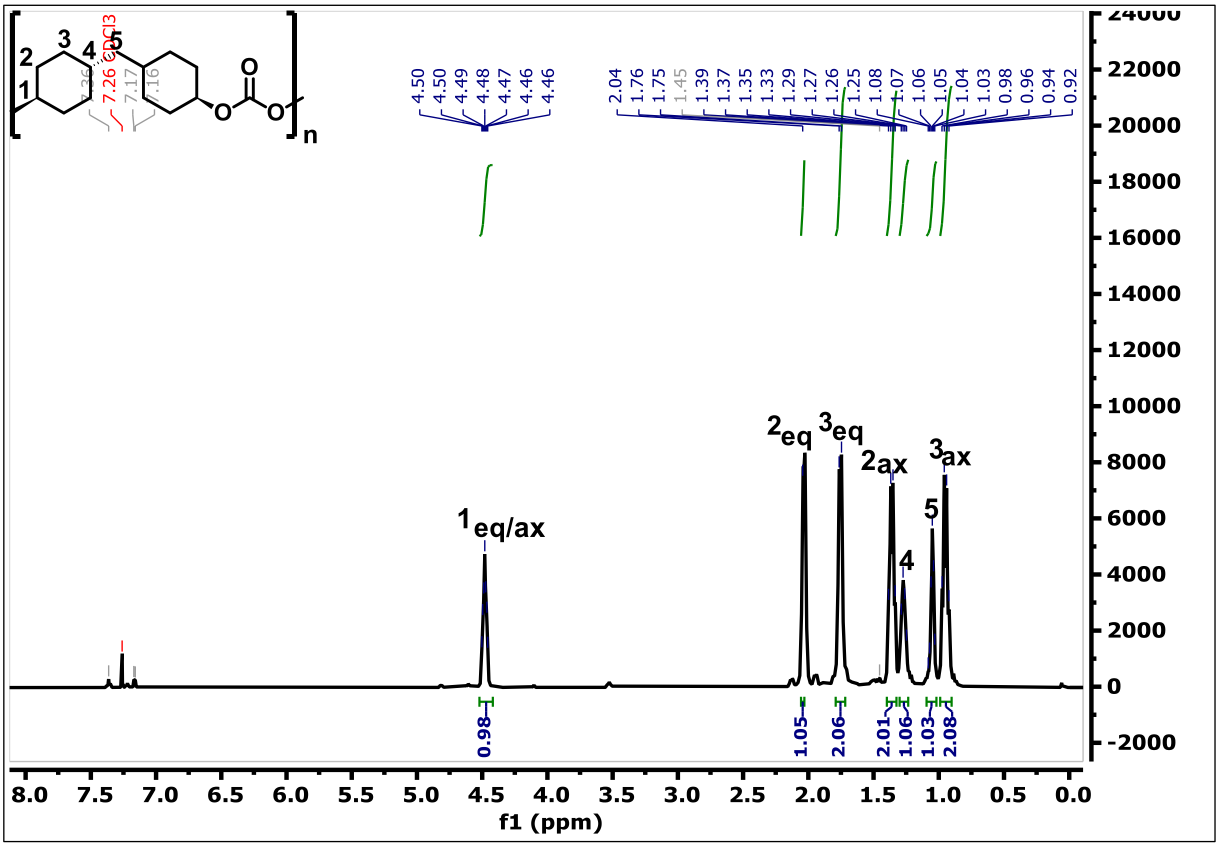

Figure S29 ^{1}H spectrum of PC-MBC$_{trans\text{-}trans}$

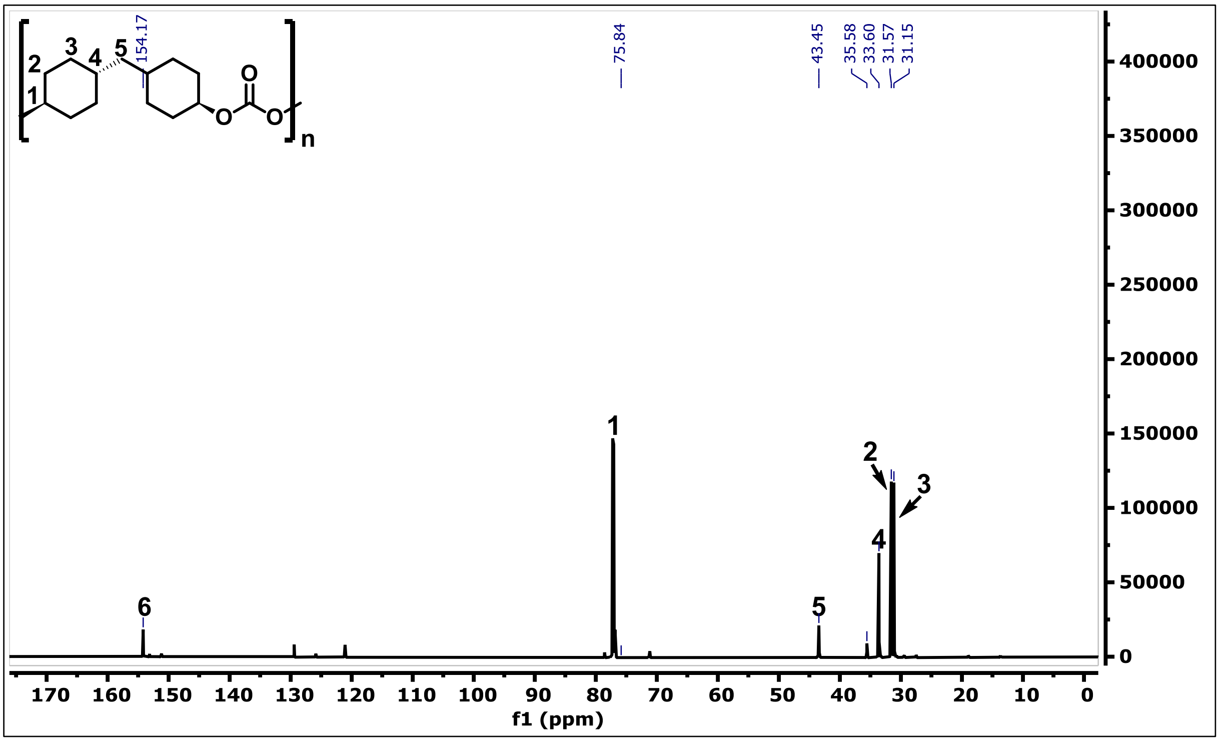

Figure S30 ^{13}C spectrum of PC-MBC$_{trans\text{-}trans}$

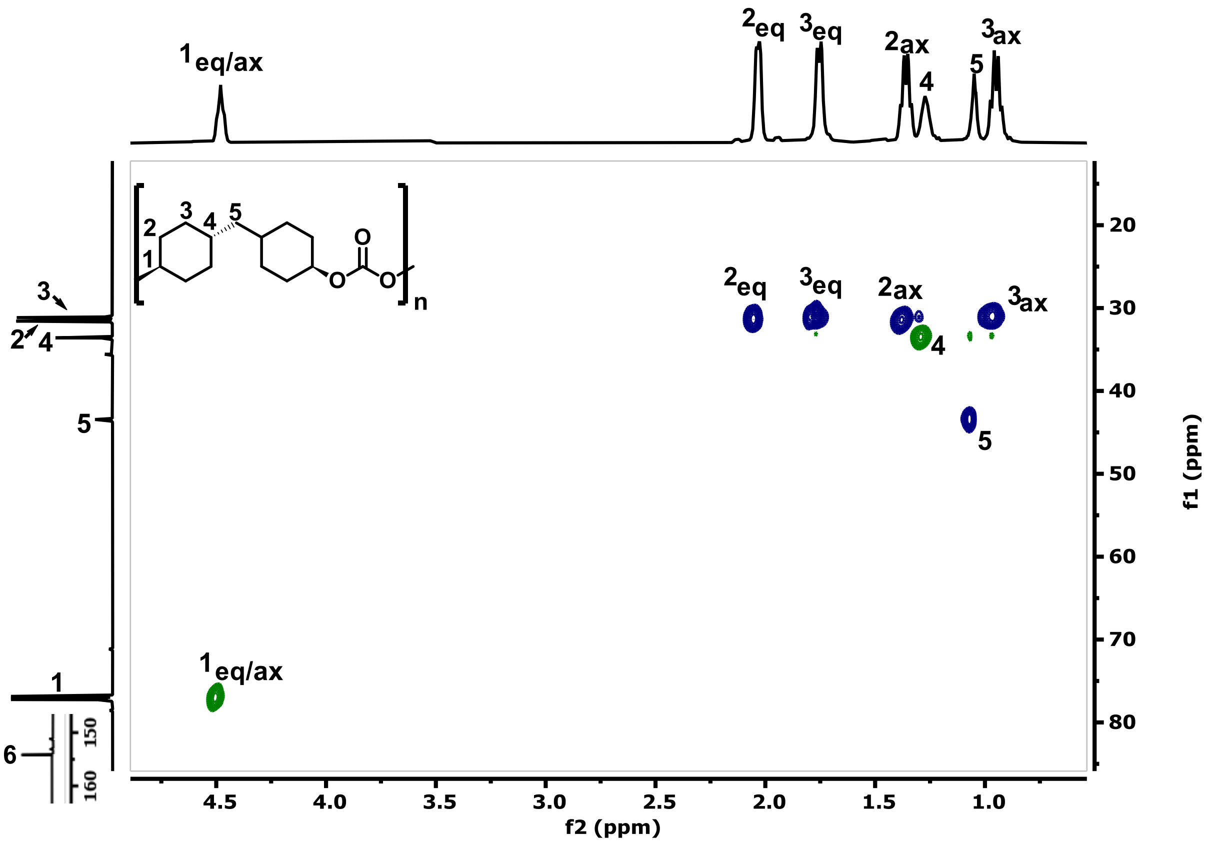

Figure S31 HSQC spectrum of PC-MBC$_{\text{trans-trans}}$

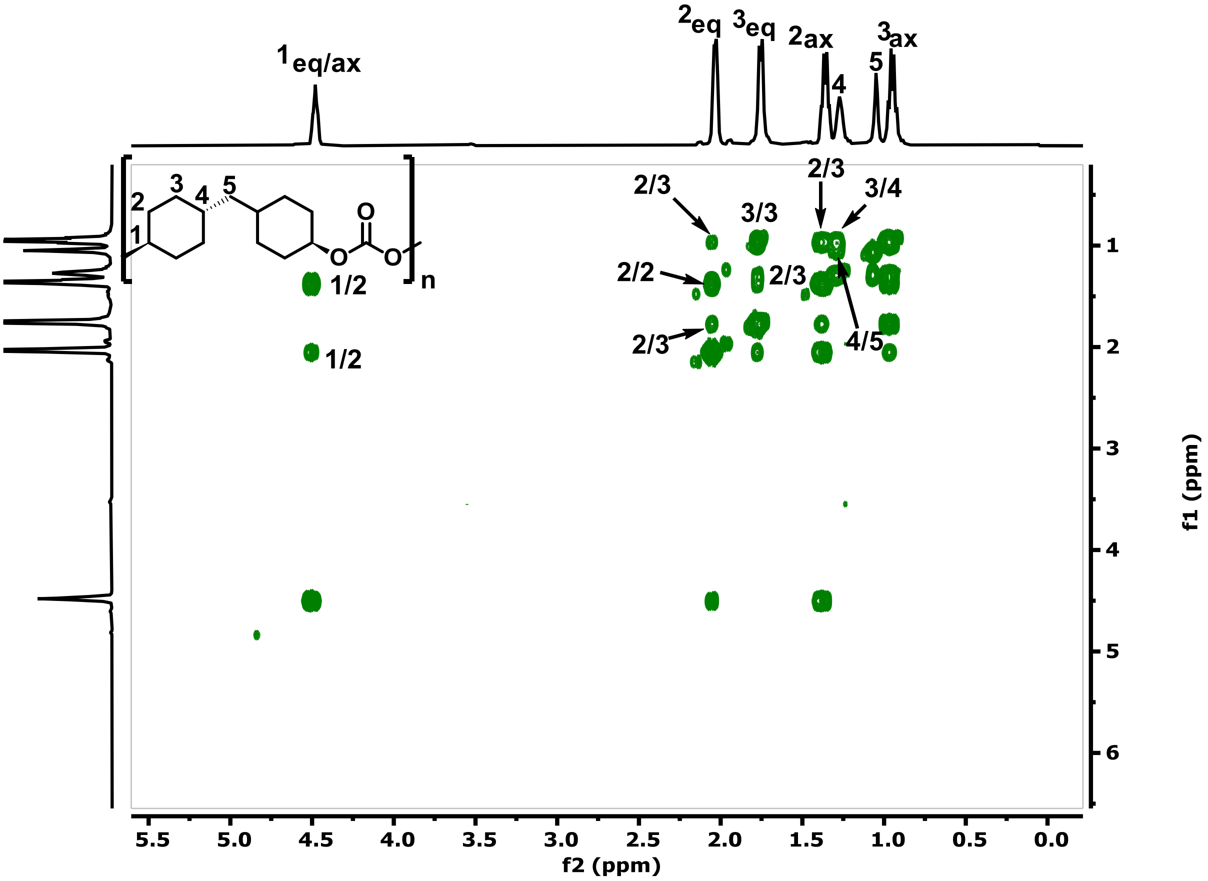

Figure S32 COSY spectrum of PC-MBC$_{\text{trans-trans}}$

3.2 TGA plots of PC-MBC$_{cis\text{-}cis}$, PC-MBC$_{cis\text{-}trans}$, PC-MBC$_{trans\text{-}trans}$ and PC-MBC$_{mixture}$

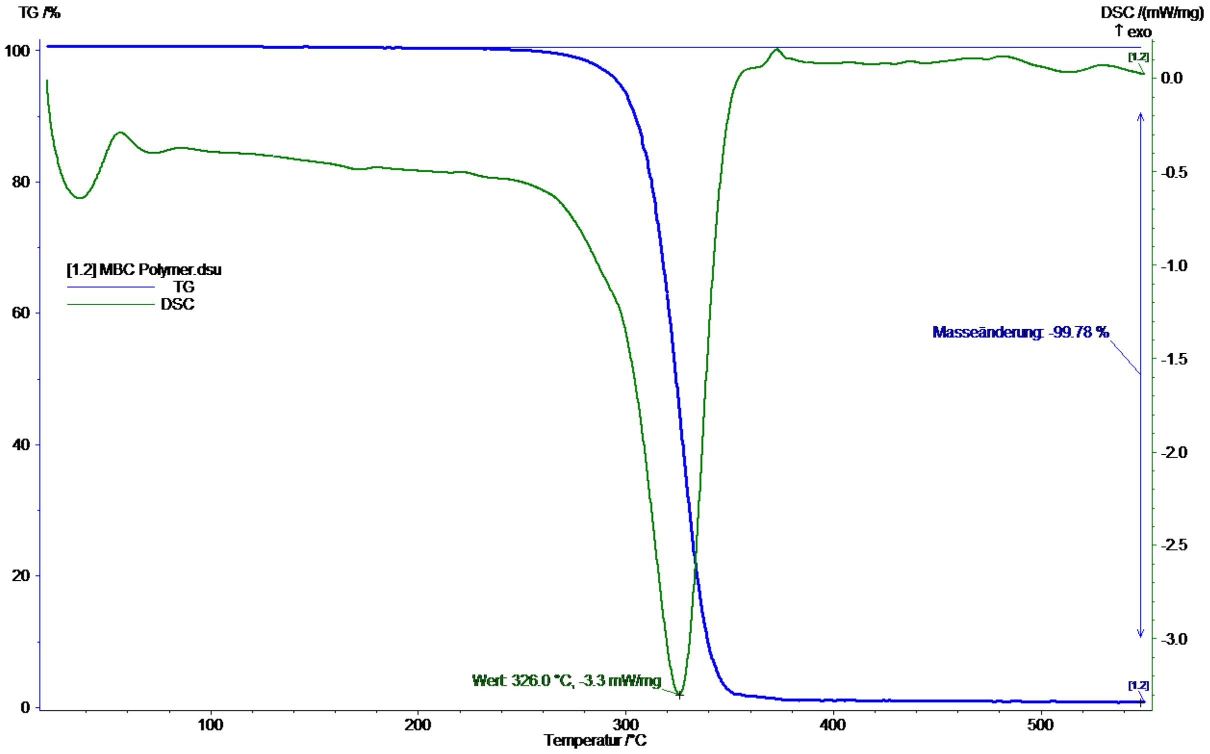

Figure S33 TGA plot of PC-MBC$_{mixture}$

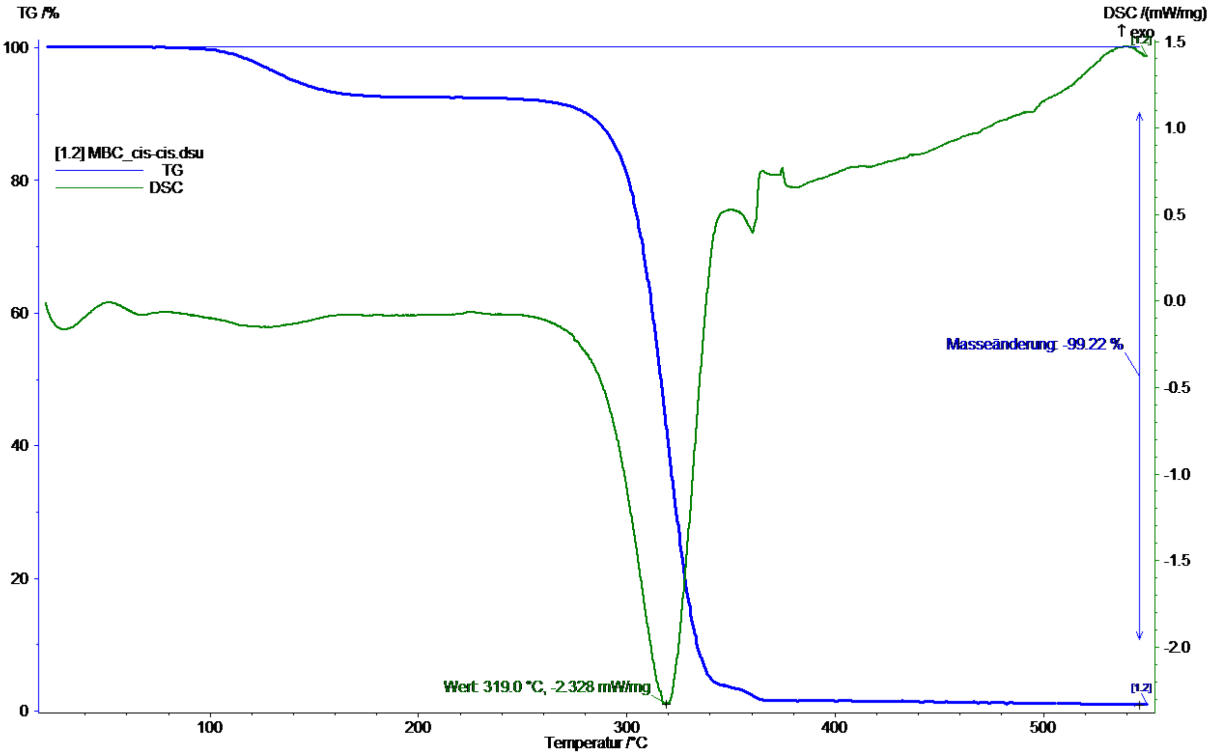

Figure S34 TGA plot of PC-MBC$_{cis\text{-}cis}$

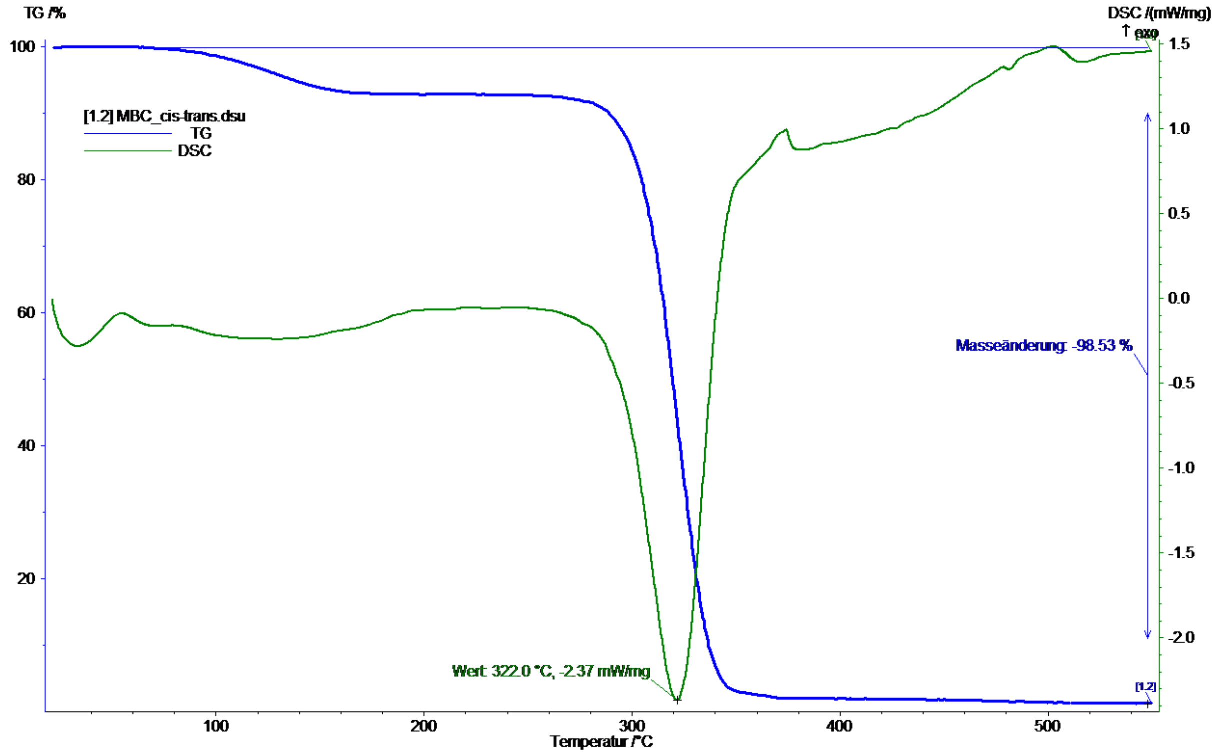

Figure S35 TGA plot of PC-$MBC_{cis\text{-}trans}$

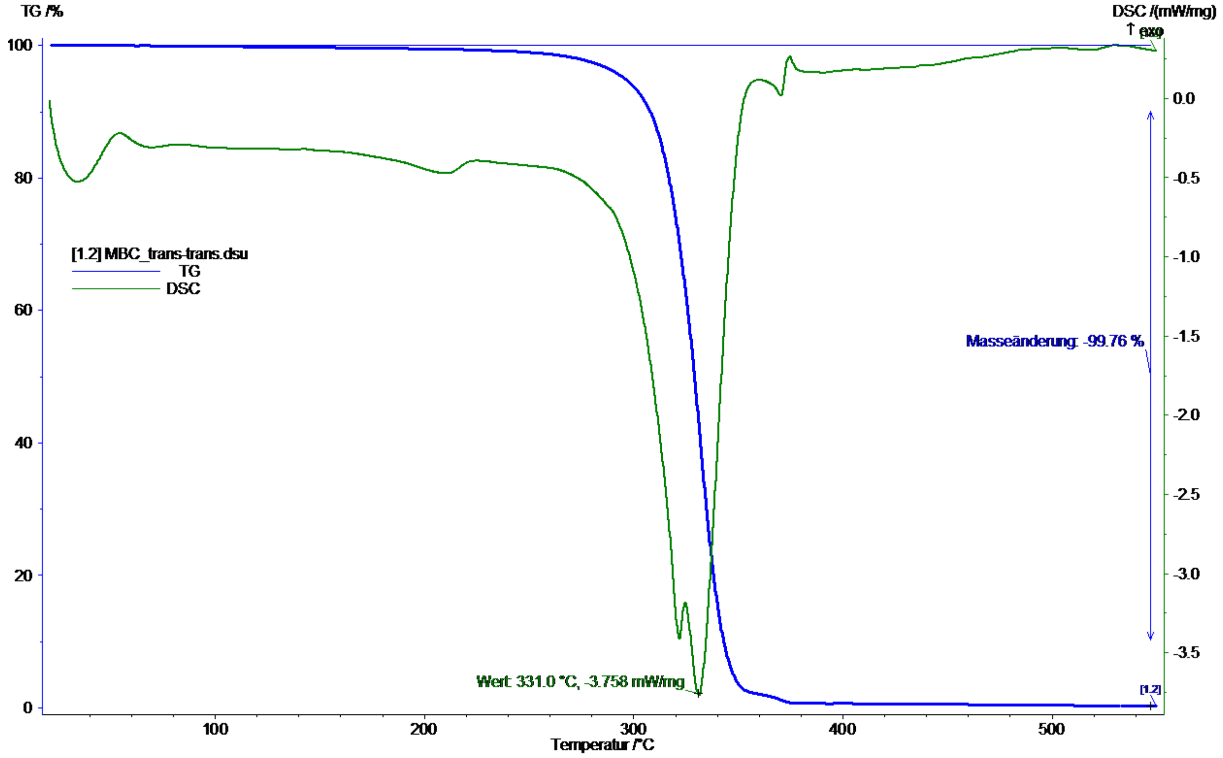

Figure S36 TGA plot of PC-$MBC_{trans\text{-}trans}$

3.3 DSC traces of PC-MBC$_{cis\text{-}cis}$, PC-MBC$_{cis\text{-}trans}$, PC-MBC$_{trans\text{-}trans}$ and PC-MBC$_{mixture}$

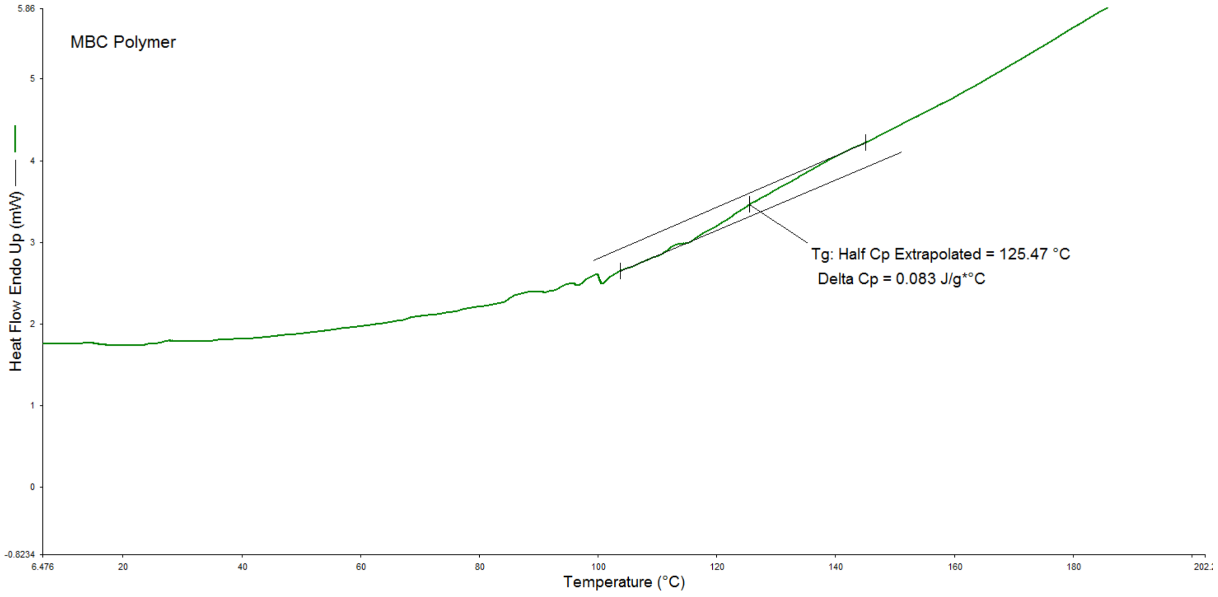

Figure S37 DSC trace of PC-MBC$_{mixture}$

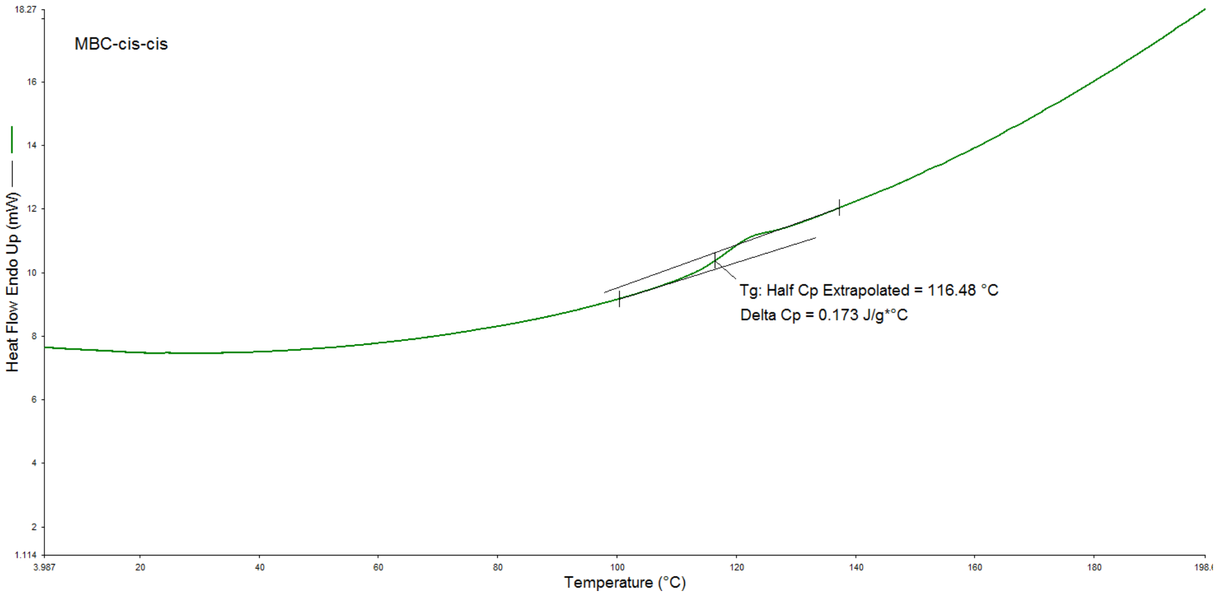

Figure S38 DSC trace of PC-MBC$_{cis\text{-}cis}$

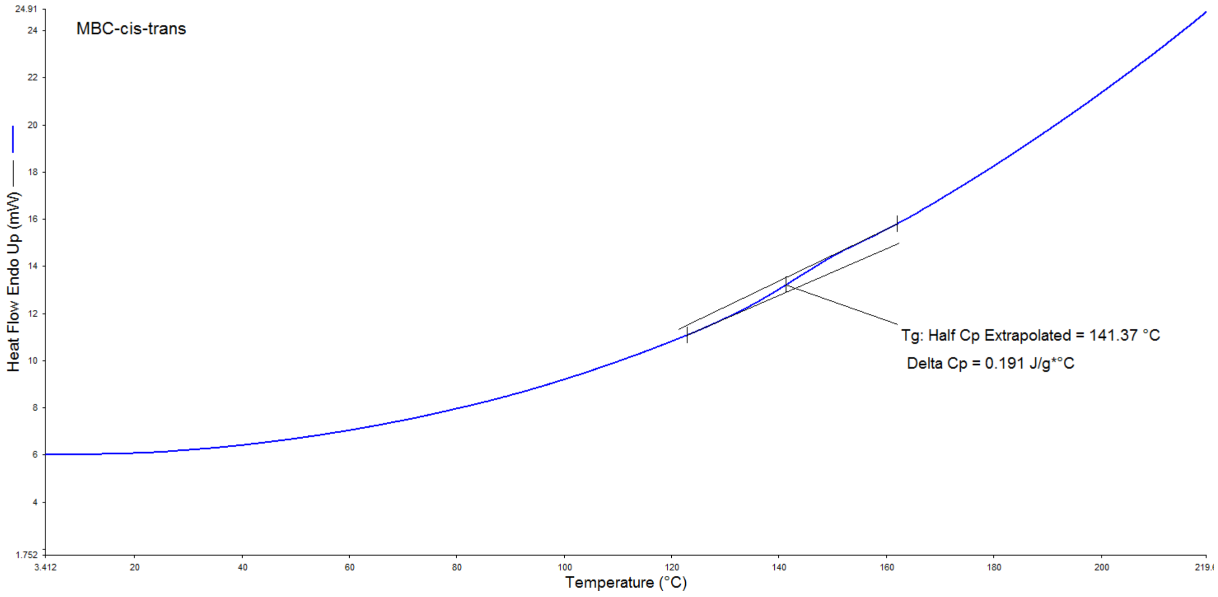

Figure S39 DSC trace of PC-$MBC_{cis\text{-}trans}$

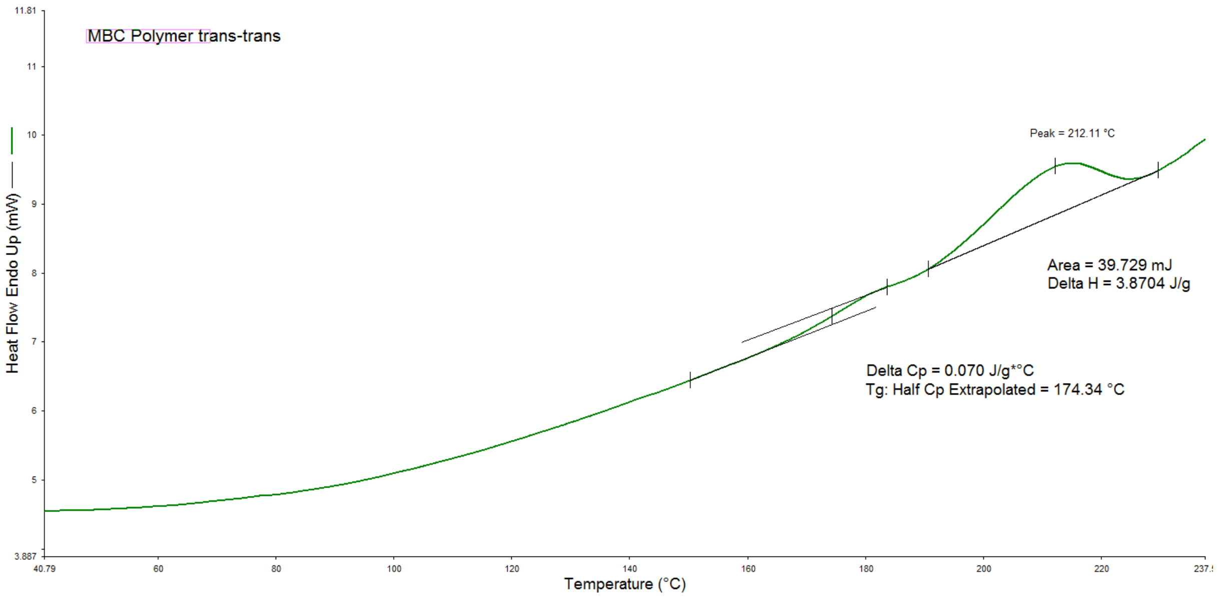

Figure S40 DSC trace of PC-$MBC_{trans\text{-}trans}$

3.4 GPC traces of PC-MBC$_{cis\text{-}cis}$, PC-MBC$_{cis\text{-}trans}$, PC-MBC$_{trans\text{-}trans}$ and PC-MBC$_{mixture}$

Acquired by	: System Administrator
Sample Name	: MBC cis-cis
Sample ID	:
Vail#	: 2
Injection Volume	: 100 uL
Data Filename	: MZ_Linear_CHCl3_062022_kb_MBC cis-cis_002.lcd
Method Filename	: MZ_Linear_CHCl3_062022_kb.lcm
Batch Filename	: Batch9.lcb
Report Filename	: GPC_Report.lsr
Date Acquired	: 28.06.2022 12:03:08
Data Processed	: 28.06.2022 12:39:10

Chromatogram & Calibration Curve

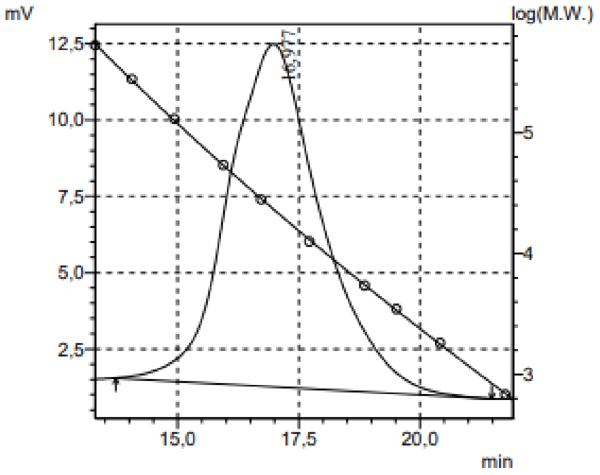

Molecular Weight Distribution Curve

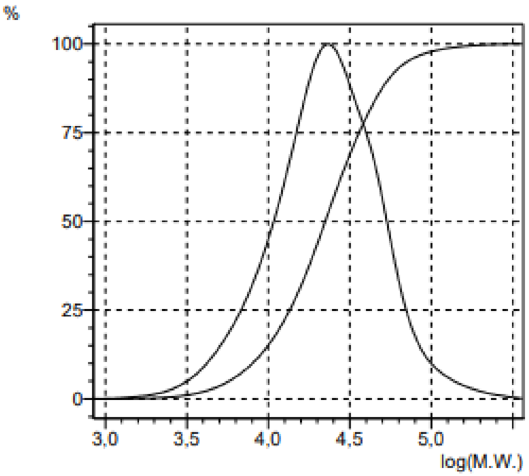

GPC Calculation Results

Peak#:1 (Detector B Channel 1)

[Peak Information]

	Time(min)	Volume(mL)	Molecular Weight	Height
Start	13,725	13,725	366595	1545
Top	16,977	16,977	23276	11229
End	21,483	21,483	842	865

Area : 1496986
Area% : 100,0000

[Average Molecular Weight]

Number Average Molecular Weight(Mn)	15963
Weight Average Molecular Weight(Mw)	28948
Z Average Molecular Weight(Mz)	51808
Z+1 Average Molecular Weight(Mz1)	94457
Mw/Mn	1,81350
Mv/Mn	0,00000
Mz/Mw	1,78967

Detector B Channel 1

[Average Molecular Weight(Total)]

Number Average Molecular Weight(Mn)	15963
Weight Average Molecular Weight(Mw)	28948
Z Average Molecular Weight(Mz)	51808
Z+1 Average Molecular Weight(Mz1)	94457
Mw/Mn	1,81350
Mv/Mn	0,00000
Mz/Mw	1,78967

Figure S41 GPC trace of PC-MBC$_{cis\text{-}cis}$

Acquired by	: System Administrator
Sample Name	: MBC cis-trans
Sample ID	:
Vail#	: 3
Injection Volume	: 100 uL
Data Filename	: MZ_Linear_CHCl3_062022_kb_MBC cis-trans_003.lcd
Method Filename	: MZ_Linear_CHCl3_062022_kb.lcm
Batch Filename	: Batch9.lcb
Report Filename	: GPC_Report.lsr
Date Acquired	: 28.06.2022 12:39:40
Data Processed	: 28.06.2022 13:15:42

Chromatogram & Calibration Curve

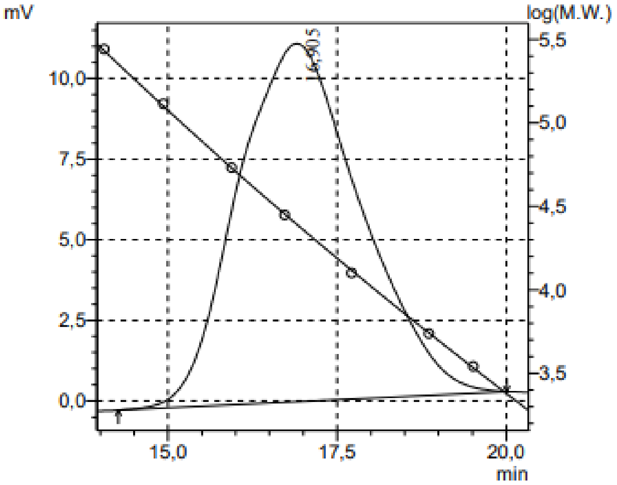

Molecular Weight Distribution Curve

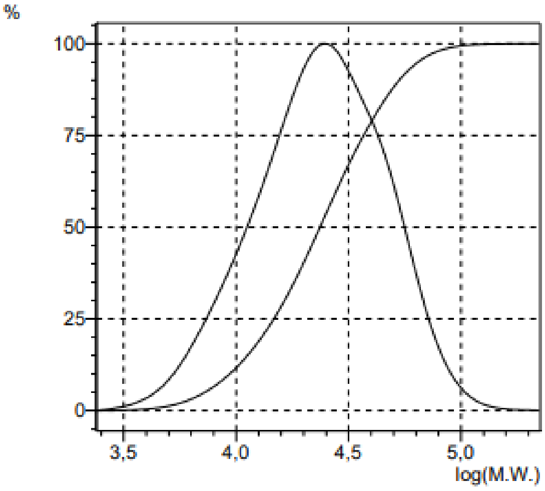

GPC Calculation Results

Peak#:1 (Detector B Channel 1)
[Peak Information]

	Time(min)	Volume(mL)	Molecular Weight	Height
Start	14,267	14,267	225377	-286
Top	16,905	16,905	24646	11105
End	20,008	20,008	2380	290

Area : 1406408
Area% : 100,0000

[Average Molecular Weight]

Number Average Molecular Weight(Mn)	18347
Weight Average Molecular Weight(Mw)	28295
Z Average Molecular Weight(Mz)	40815
Z+1 Average Molecular Weight(Mz1)	55098
Mw/Mn	1,54220
Mv/Mn	0,00000
Mz/Mw	1,44251

Detector B Channel 1
[Average Molecular Weight(Total)]

Number Average Molecular Weight(Mn)	18347
Weight Average Molecular Weight(Mw)	28295
Z Average Molecular Weight(Mz)	40815
Z+1 Average Molecular Weight(Mz1)	55098
Mw/Mn	1,54220
Mv/Mn	0,00000
Mz/Mw	1,44251

Figure S42 GPC trace of PC-MBC$_{cis\text{-}trans}$

Acquired by : System Administrator
Sample Name : MBC Polymer
Sample ID :
Vail# : 1
Injection Volume : 100 uL
Data Filename : MZ_Linear_CHCl3_062022_kb_MBC Polymer_001.lcd
Method Filename : MZ_Linear_CHCl3_062022_kb.lcm
Batch Filename : Batch9.lcb
Report Filename : GPC_Report.lsr
Date Acquired : 28.06.2022 11:26:35
Data Processed : 28.06.2022 12:02:37

Chromatogram & Calibration Curve

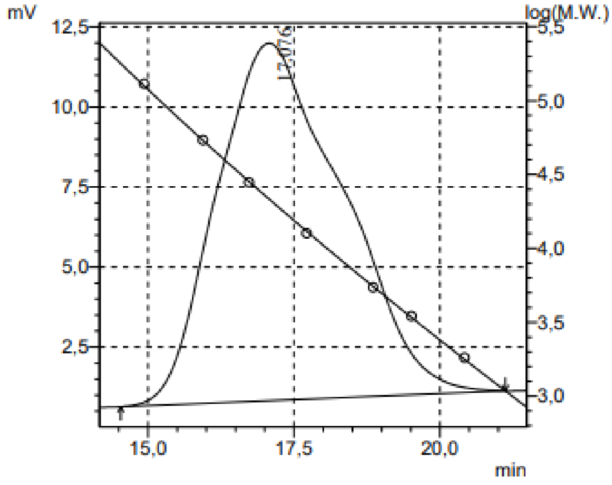

Molecular Weight Distribution Curve

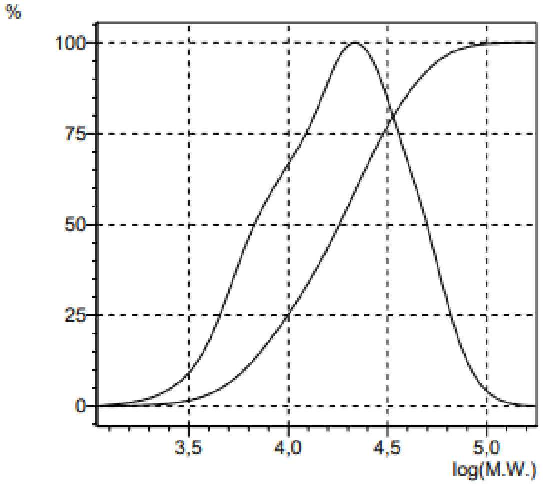

GPC Calculation Results

Peak#:1 (Detector B Channel 1)
[Peak Information]

	Time(min)	Volume(mL)	Molecular Weight	Height
Start	14,533	14,533	178144	636
Top	17,076	17,076	21528	11167
End	21,117	21,117	1086	1130

Area : 1670825
Area% : 100,0000

[Average Molecular Weight]

Number Average Molecular Weight(Mn)	12628
Weight Average Molecular Weight(Mw)	22695
Z Average Molecular Weight(Mz)	35820
Z+1 Average Molecular Weight(Mz1)	49805
Mw/Mn	1,79724
Mv/Mn	0,00000
Mz/Mw	1,57833

Detector B Channel 1
[Average Molecular Weight(Total)]

Number Average Molecular Weight(Mn)	12628
Weight Average Molecular Weight(Mw)	22695
Z Average Molecular Weight(Mz)	35820
Z+1 Average Molecular Weight(Mz1)	49805
Mw/Mn	1,79724
Mv/Mn	0,00000
Mz/Mw	1,57833

Figure S43 GPC trace of PC-MBC$_{mixture}$

Acquired by	: System Administrator
Sample Name	: MBC trans-trans_
Sample ID	:
Vail#	: 1
Injection Volume	: 100 uL
Data Filename	: MZ_Linear_CHCl3_062022_kb_MBC trans-trans__001.lcd
Method Filename	: MZ_Linear_CHCl3_062022_kb.lcm
Batch Filename	: Batch9.lcb
Report Filename	: GPC_Report.lsr
Date Acquired	: 28.06.2022 14:16:01
Data Processed	: 28.06.2022 14:52:03

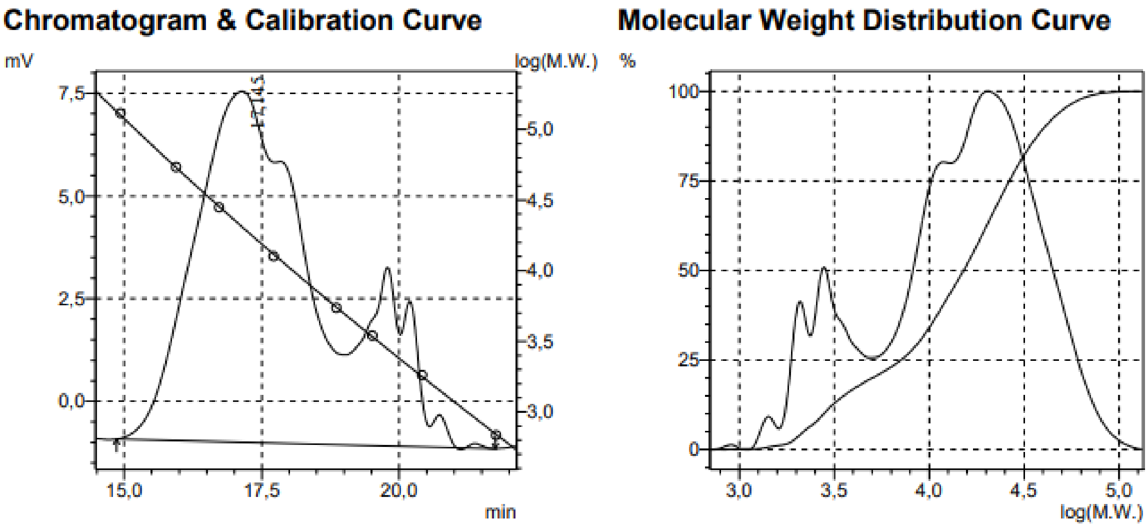

GPC Calculation Results

Peak#:1 (Detector B Channel 1)
[Peak Information]

	Time(min)	Volume(mL)	Molecular Weight	Height
Start	14,858	14,858	134249	-920
Top	17,145	17,145	20380	8543
End	21,750	21,750	700	-1154

Area : 1388186
Area% : 100,0000

[Average Molecular Weight]

Number Average Molecular Weight(Mn)	7863
Weight Average Molecular Weight(Mw)	19169
Z Average Molecular Weight(Mz)	32669
Z+1 Average Molecular Weight(Mz1)	45134
Mw/Mn	2,43803
Mv/Mn	0,00000
Mz/Mw	1,70423

Detector B Channel 1
[Average Molecular Weight(Total)]

Number Average Molecular Weight(Mn)	7863
Weight Average Molecular Weight(Mw)	19169
Z Average Molecular Weight(Mz)	32669
Z+1 Average Molecular Weight(Mz1)	45134
Mw/Mn	2,43803
Mv/Mn	0,00000
Mz/Mw	1,70423

Figure S44 GPC trace of PC-$MBC_{trans\text{-}trans}$

Supplementary references

1. X. Y. Wu, M. V. Galkin, K. Barta, *Chem. Catal.*, 2021, **1**, 1360-1362.
